# Intercepting the Gold‐Catalysed Meyer–Schuster Rearrangement by Controlled Protodemetallation: A Regioselective Hydration of Propargylic Alcohols

**DOI:** 10.1002/adsc.201600101

**Published:** 2016-04-27

**Authors:** Matthew N. Pennell, Michael P. Kyle, Samantha M. Gibson, Louise Male, Peter G. Turner, Richard S. Grainger, Tom D. Sheppard

**Affiliations:** ^1^ Department of Chemistry, University College London Christopher Ingold Laboratories 20 Gordon Street London WC1H 0AJ U.K.; ^2^ School of Chemistry University of Birmingham Edgbaston, Birmingham B15 2TT U.K.; ^3^ GlaxoSmithKline Medicines Research Centre Gunnels Wood Road Stevenage, Herts SG1 2NY U.K.

**Keywords:** aldol reaction, alkynes, boronic acids, enolates, gold

## Abstract

The regioselective gold‐catalysed hydration of propargylic alcohols to β‐hydroxy ketones can be achieved by diverting the gold‐catalysed Meyer–Schuster rearrangement through the addition of a protic additive with a p*K*_a_ of 7–9 such as *p*‐nitrophenol, boric acid or a boronic acid. This provides an interesting alternative to an aldol reaction when combined with the straightforward addition of an alkyne to an aldehyde or ketone. The gold‐catalysed reaction of an electron‐deficient, sterically hindered propargylic alcohol with a boronic acid led to the formation of an unusually stable cyclic boron enolate.

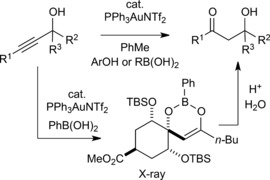

Propargylic alcohols are versatile compounds which can be used in a wide variety of gold‐catalysed transformations.^1^ The gold‐catalysed Meyer–Schuster rearrangement of propargylic alcohols is a well‐established method for the efficient preparation of a wide variety of enones.^2^,^3^ It is also possible to intercept the Meyer–Schuster rearrangement with a variety of electrophiles to give access to α‐haloenones,^4^ α‐arylenones,^5^ or α,α‐diiodo‐β‐hydroxy ketones.^6^ In this update, we describe our studies on the use of controlled protodemetallation to divert the gold‐catalysed Meyer–Schuster rearrangement to give access to β‐hydroxy ketones and related derivatives.

In 2010, we reported that the gold‐catalysed cyclisation of *o*‐alkynlbenzeneboronic acids could be used as an extremely mild method to generate boron enolates **2** (Scheme 1, **A**).^7^ In an attempt to extend this chemistry, we envisaged that propargylic alcohols **3** would react with boronic acids to give half esters **4**, which could undergo cyclisation to the corresponding boron enolate **5**. In the event, these reactions led almost exclusively to the formation of Meyer–Schuster products **6**. We observed, however, that a protic additive such as an alcohol or boronic acid served to accelerate the gold‐catalysed Meyer–Schuster rearrangement of propargylic alcohols **3** to enones **6** in a non‐polar organic solvent such as toluene (Scheme 1, **B** and **C**).^3^ During the course of our study, we found that in the presence of a catalytic quantity of boronic acid the β‐hydroxy ketone **7a** was observed as a significant by‐product in reactions of some secondary propargylic alcohols (Scheme 2). When methanol was used as the additive with the same substrates, the sole product was the enone **6a**, and the formation of **7a** was completely suppressed.

**Scheme 1:**
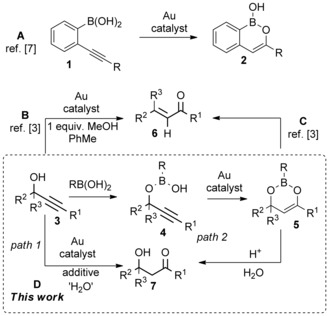
Gold‐catalysed addition of boronic acids to alkynes^7^ and the gold‐catalysed Meyer–Schuster rearrangement in the presence of protic additives.^3^

**Scheme 2:**
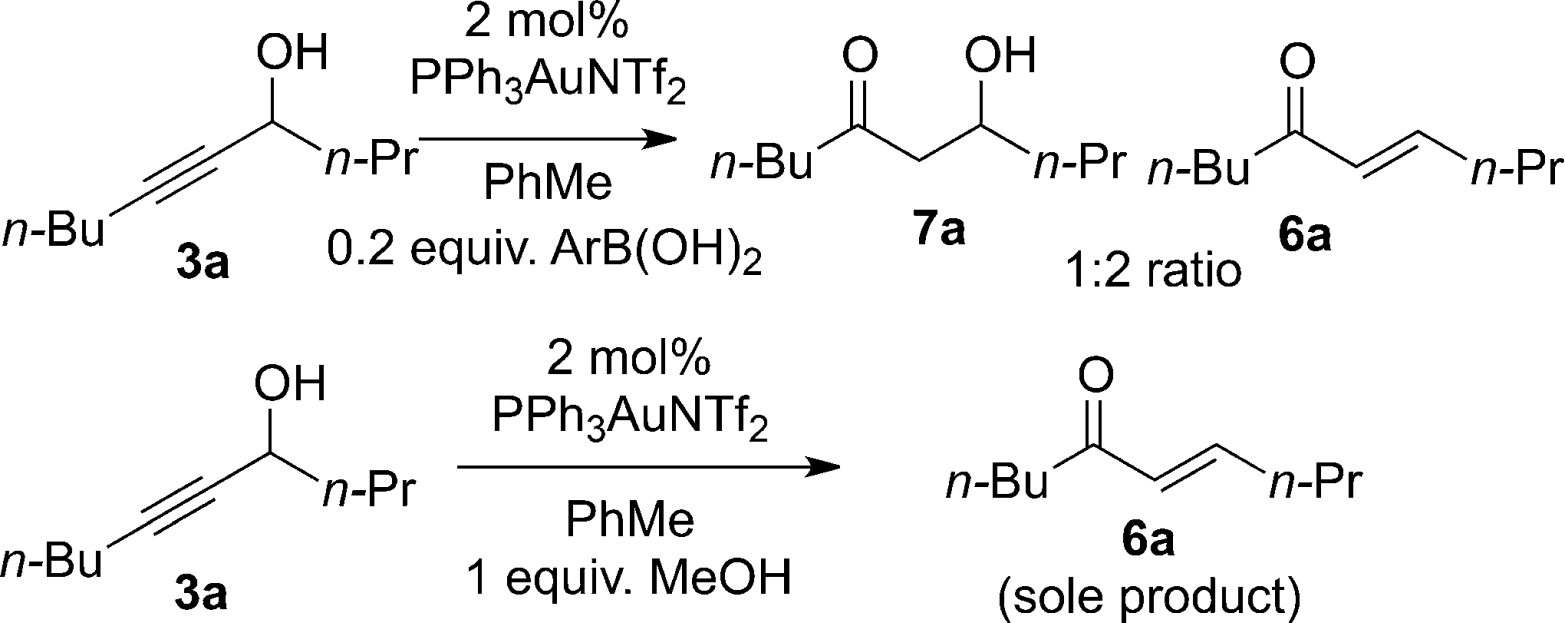
Gold‐catalysed Meyer–Schuster rearrangement of propargylic alcohol **3a** in the presence of protic additives.

We envisaged that a regioselective hydration of a propargylic alcohol to a ketone (Scheme 1, **D**) could provide an interesting alternative to a traditional aldol reaction between a ketone and an aldehyde. This would circumvent the requirement to use low temperatures and stoichiometric reagents to achieve regioselective enolisation of the ketone, prior to addition of the aldehyde. Whilst isolated examples of gold‐ or mercury‐catalysed hydration reactions of propargylic alcohols have been described,^8^,^9^ these reports largely involve terminal alkynes^8^ or the formation of β‐hydroxy ketones as by‐products.^9^ To the best of our knowledge, the gold‐catalysed regioselective hydration of propargylic alcohols containing an internal alkyne has not been described.^10^,^11^

We hypothesised that through suitable selection of a protic additive, the Meyer–Schuster pathway could be diverted either to give β‐hydroxy ketone **7** directly (Scheme 1, path 1), or to generate the boron enolate **5** which could yield **7** *via* hydrolysis (path 2). We therefore set out to investigate in more detail the effect of the choice of protic additive on the product distribution of the reaction, with the aim of identifying conditions which would give the β‐hydroxy ketone **7a** as the major product (Scheme 3 and Table 1). In the absence of an additive, almost equal quantities of the β‐hydroxy ketone **7a** and the enone **6a** were obtained (entry 1). Interestingly, the outcome of the reaction appeared to correlate reasonably well with the p*K*_a_ of the protic additive used (entries 2–11). As the acidity of the additive increased (Table 1, entries 2–7), increasing amounts of the direct hydration product **7a** were observed, with this compound being the major reaction product when 4‐nitrophenol (PNPOH) was used. However, as the acidity of the additive was increased further (entries 8–11), the product ratio reversed with enone **6a** once again becoming the major product. This suggests that there is an optimum ‘p*K*_a_ window’ in which the formation of **7a** is favoured. With this information in hand, we went on to examine these conditions for the regioselective hydration of a selection of different propargylic alcohols (Scheme 4).

**Scheme 3:**
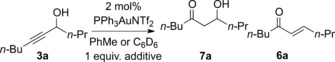
Investigation of the effect of additive acidity on the ratio of **7a**:**6a**.

**Table 1:** The effect of different additives on the gold‐catalysed reaction of propargylic alcohol **3a**.

Entry	Additive	p*K*_a_ (H_2_O)	Conv.^[a]^	Ratio of **7a**:**6a** ^[b]^
1	none	–	64%	1:1.1
2	MeOH	15.7	100%	**6a** only
3	CF_3_CH_2_OH	12.5	59%	1:1
4	PhOH	10.0	100%	2:3
5	B(OH)_3_	9.2	87%	2:3
6	PMPB(OH)_2_^[c]^	8–9	99%	1:2
**7**	**PNPOH** ^[d]^	**7.1**	**100%**	**3:1**
8	C_6_F_5_OH	5.5	61%	1:2
9	C_6_Cl_5_OH	4.7	93%	1:1
10	AcOH	4.7	68%	2:3
11	CF_3_CO_2_H	0.3	100%	1:3

^[a]^ Conversion of **3a** to **7a**/**6a** as measured by crude ^1^H NMR. ^[b]^ Determined from the crude ^1^H NMR. ^[c]^ PMP=4‐MeOC_6_H_4_. ^[d]^ PNP=4‐O_2_NC_6_H_4_.

**Scheme 4:**
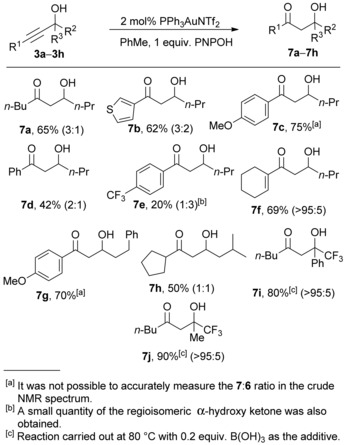
Scope of the regioselective hydration reaction. Isolated yields shown; the ratio of **7**:**6**, measured by crude ^1^H NMR, is shown in parentheses.

Pleasingly, under these conditions the β‐hydroxy ketone **7a** was obtained in 65% isolated yield, with the remaining balance of the material being the corresponding enone **6a**. The hydration reaction proceeded with very high regioselectivity and no trace of the isomeric α‐hydroxy ketone was observed.^11^ Aryl ketones **7b**–**7e**, including a heterocyclic example **7b**, were obtained in variable yields, depending upon the electronics of the aromatic ring. Whilst the hydration reactions to generate electron‐rich ketones **7b** and **7c** proved to be particularly effective, phenyl ketone **7d** and electron‐deficient aryl ketone **7e** were obtained in lower yields. In the latter case, the enone **6e** was the major product from the reaction and in contrast to all the other reactions studied, small quantities of the isomeric α‐hydroxy ketone were isolated. Pleasingly, an enone could also be obtained effectively through regioselective hydration of a conjugated enyne (**7f**). Hydration of propargylic alcohols **3g** and **3h**, to give aryl β‐hydroxy ketone **7g** and branched alkyl ketone **7h**, respectively, also proceeded smoothly. Initial attempts to achieve regioselective hydration of the fluorinated tertiary propargylic alcohols **3i** and **3j** were unsuccessful under the standard conditions as no reaction was observed. Higher conversions were seen upon heating the reaction mixture, but complex mixtures of products were observed with PNPOH as the additive. However, by using boric acid, the fluorinated ketones **7i** and **7j** were obtained in excellent yields from the direct hydration reaction, and in these cases no traces of the Meyer–Schuster rearrangement products **6i**/**6j** were observed. Other tertiary propargylic alcohols such as **3k**, derived from pinacolone, failed to give any β‐hydroxy ketone with the enone **6k** being observed as the sole product (Scheme 5). Similarly, aryl‐substituted propargylic alcohols **3l**–**3n** yielded only the corresponding enones **6l**–**6n**, regardless of whether the aryl group was unsubstituted, electron‐rich or electron‐deficient.

**Scheme 5:**
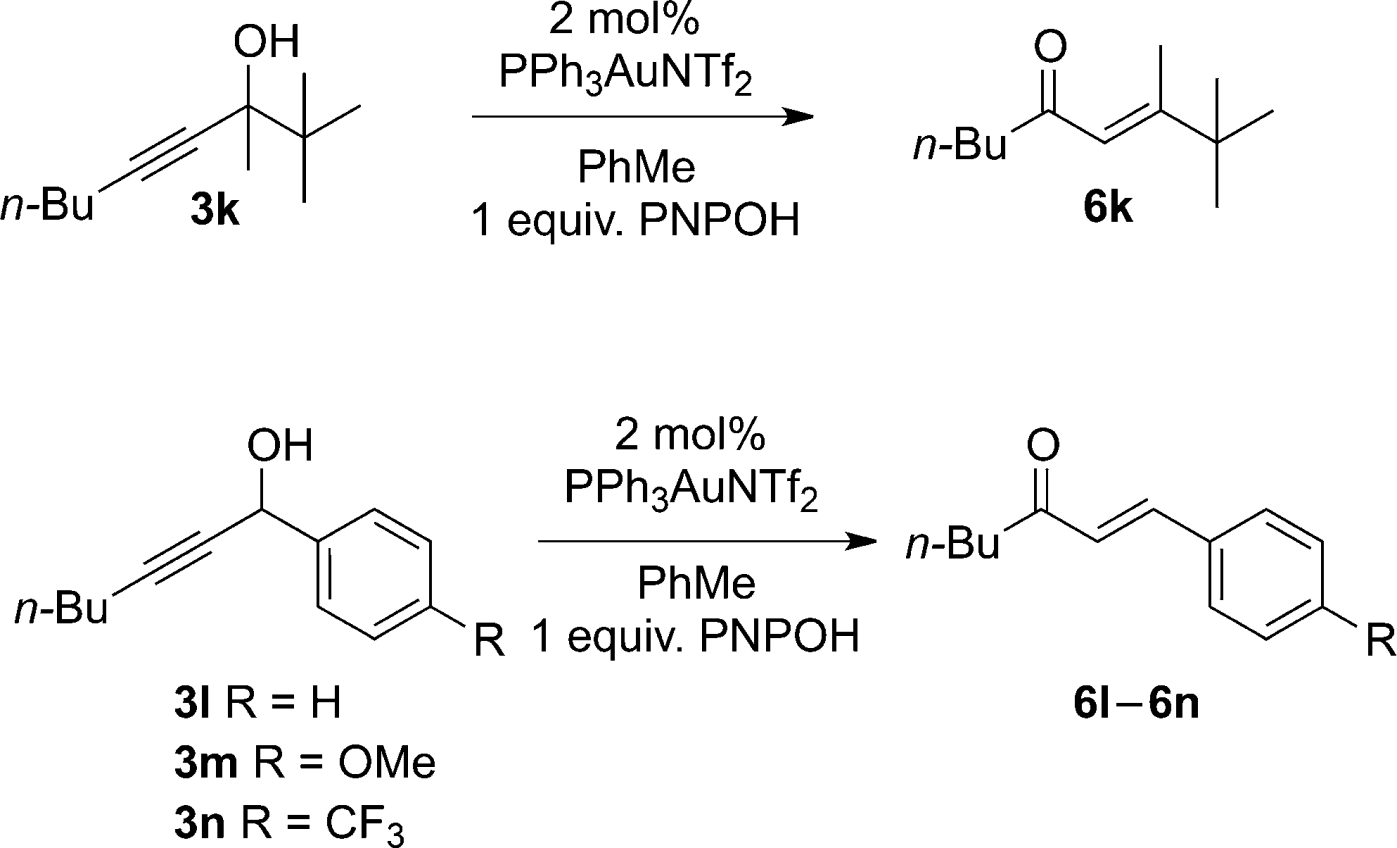
Attempted hydration of tertiary propargylic alcohol **3k** and aryl‐substituted propargylic alcohols **3l**–**3n**.

The formation of β‐hydroxy ketones **7** by regioselective hydration of propargylic alcohols offers an intriguing alternative to the aldol reaction of a ketone and an aldehyde. In order to further demonstrate the potential of this approach, we subjected enantioenriched propargylic alcohol **3o**, prepared using Carreira's asymmetric addition method,^12^ to the hydration reaction. Pleasingly, the corresponding β‐hydroxy ketone **7o** was obtained in 65% yield with complete retention of enantiopurity (Scheme 6).

**Scheme 6:**
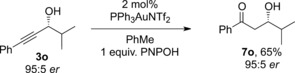
Regioselective hydration of an enantioenriched propargylic alcohol.

In most of the hydration reactions, the formation of the desired β‐hydroxy ketone (**7a**–**7h**, **7o**) was accompanied by the formation of smaller quantities of the Meyer–Schuster rearrangement product (**6a**–**6h**, **6o**). Interestingly, under the hydration reaction conditions, the conversion of the starting material **3** into both products **6** and **7** was considerably slower than the Meyer–Schuster rearrangement to give enone **6** in the presence of methanol as an additive.^13^ This suggests that the PNPOH additive is able to suppress the Meyer–Schuster rearrangement pathway that operates in the presence of MeOH. The formation of the enone by‐product in these hydration reactions appears to be strongly influenced by the electronic environment around the alcohol group. Substrates containing strongly electron‐withdrawing substituents close to the alcohol (e.g., CF_3_) did not readily form the enone at all, whereas propargylic alcohols which contained groups able to stabilise a positive charge at the alcohol carbon (tertiary propargylic alcohols, aryl‐substituted secondary propargylic alcohols) tended to form exclusively the enone **6**. We therefore hypothesised that under these conditions, enone formation must proceed *via* a mechanism that involves an intermediate with substantial cationic character at the alcohol carbon. Coupled with the fact that the regioselective hydration reactions in Scheme 4 were generally much slower than the direct Meyer–Schuster rearrangements reported previously, this suggests that the formation of the by‐product **6** may take place *via* elimination of water from the desired β‐hydroxy ketone product **7**. Resubmission of a purified sample of hydroxy ketone **7a** to the reaction conditions (Scheme 7a) led to approximately 30% conversion into the corresponding enone **6a**, suggesting that, in the presence of PNPOH as the additive, the majority of the enone by‐product observed is formed *via* an acid‐catalysed elimination of water from **7a**. This is also consistent with the observation that additives that were more acidic than PNPOH led to increased formation of enone **6a** (Table 1). Resubmission of alcohol **7a** to the Meyer–Schuster rearrangement conditions^3^ (1 equiv. of MeOH as the additive, Scheme 7b) did not lead to any elimination of water to give enone **6a**.

**Scheme 7:**
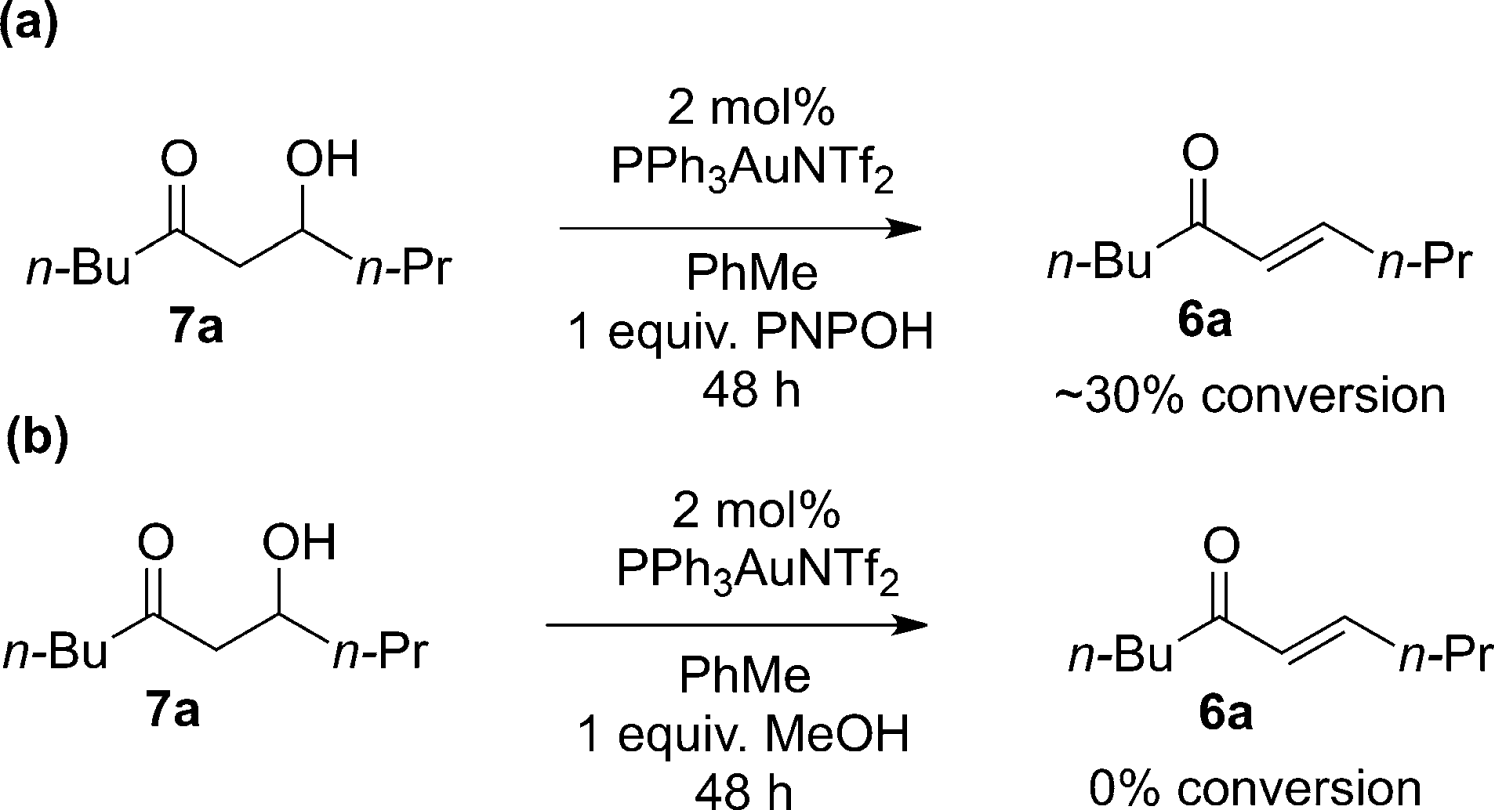
Resubmission of alcohol **7a** to the reaction conditions.

We believe that the protic additive^14^ plays multiple roles in the reaction: (i) inhibition of the ‘normal’ Meyer–Schuster pathway which gives the enone **6**; (ii) promotion of an alternative pathway leading directly to the β‐hydroxy ketone **7**; (iii) Brønsted acid catalyst for the conversion of **7** into **6** *via* elimination of water; and (iv) solubilisation of water in the non‐polar organic solvent.^15^ We propose that the most important role of the acidic additive is to promote protodeauration of the likely vinylgold intermediate **8**, generated by gold‐catalysed addition of water or the protic additive^16^ across the alkyne (Scheme 8). If ROH is sufficiently acidic, rapid protodemetallation to give intermediate **9** takes place, followed by tautomerisation or hydrolysis to provide β‐hydroxy ketone **7**. This then undergoes slow acid‐catalysed elimination of water (either *via* an enol‐assisted elimination of water under general acid catalysis **10**, or *via* an E1 mechanism depending on the substrate structure). If ROH is not sufficiently acidic (MeOH), then intermediate **8** can evolve *via* proton transfer and then elimination of water, accompanied by cleavage of the carbon‐gold bond, to give allene **12**, which undergoes tautomerisation or hydrolysis to provide **6**. Again, the elimination step (**11** to **12**) must involve a transition state with considerably positive charge build up at the propargylic alcohol carbon, as enone formation was entirely suppressed in the case of strongly electron‐deficient propargylic alcohols (**3i** and **3j**) The hypothesis that intermediate **8** should undergo protodemetallation with PNPOH is further supported by the fact that phenols (but not alcohols) have been shown to be capable of protonating an *sp* ^2^ aryl‐gold bond.^17^ It should also be noted that the propargylic alcohol **3** (and indeed the β‐hydroxy ketone **7**) can also act as a nucleophile and/or proton source in the mechanism.

**Scheme 8:**
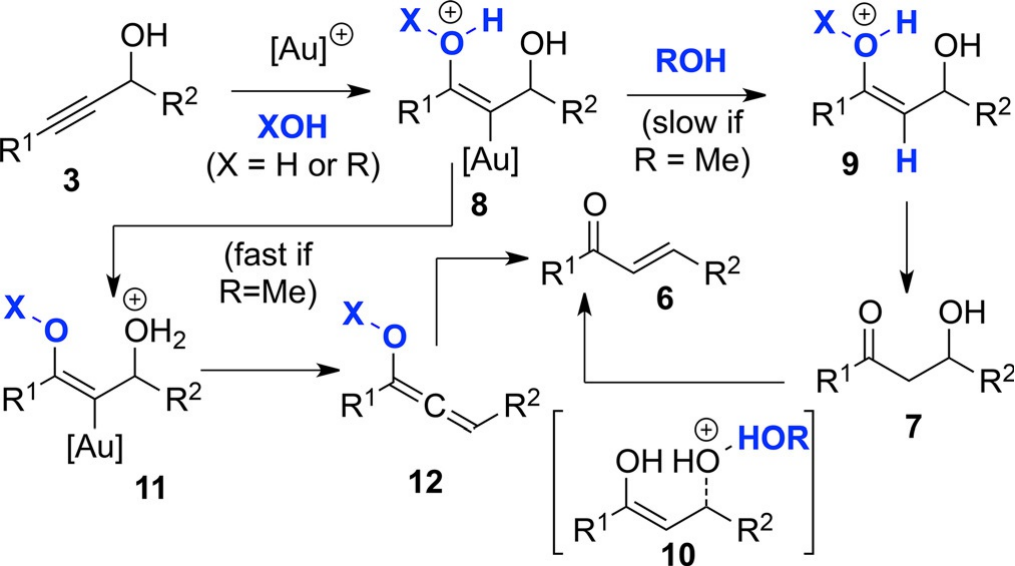
Proposed mechanistic pathways leading to **6** and **7**.

An alternative mechanism may operate in the case of boronic (or boric) acid additives. We have previously proposed cyclic enol boronates **5** as common intermediates in the formation of the enone **6** and β‐hydroxy ketone **7** (Scheme 1). Enol boronates of the general structure **5** have rarely been reported and structural data are limited.^18^ Remarkably, however, we were recently able to isolate an unusually stable example in the course of our studies directed towards a total synthesis of phyllaemblic acid and related natural products.^19^ Reaction of sterically hindered electron‐deficient propargylic alcohol **3p** with phenylboronic acid in the presence of a gold catalyst led to the formation of boron enolate **5p**, with no evidence of further conversion to the enone **6p** or β‐hydroxy ketone **7p** under the reaction conditions (Scheme 9).

**Scheme 9:**
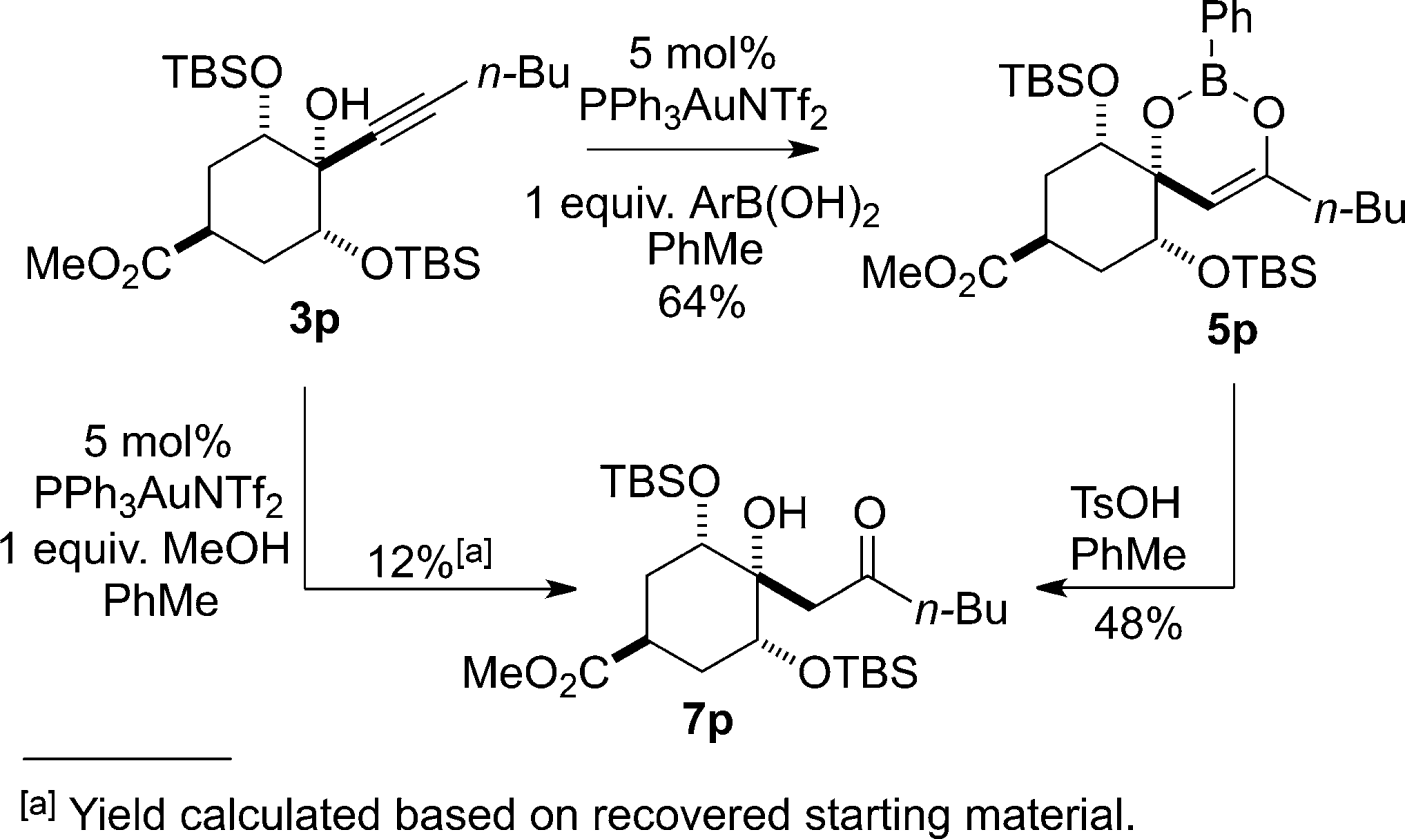
Formation of an unusually stable boron enolate **5p** *via* gold‐catalysed reaction of a hindered propargylic alcohol **3p** with phenylboronic acid.

The structure of **5p** was confirmed by single crystal X‐ray crystallography (Figure 1).^20^ The solid state structure clearly shows the planarity of the boron atom and how the two bulky TBS groups may shield both the boron atom towards nucleophilic attack and the enol double bond towards electrophilic attack. Boron enolate **5p** was exceptionally stable, being recovered unchanged after heating for 2 h at 200 °C in toluene in a microwave reactor. Treatment of **5p** with TsOH in toluene^19^ at room temperature gave a modest 48% yield of the corresponding β‐hydroxy ketone **7p**, which was also obtained in low yield as part of a complex mixture directly from propargyl alcohol **3p**, by reaction with a gold catalyst using MeOH as the protic additive. Attempted reactions of **5p** with various nucleophiles (NaOH/H_2_O_2_) or electrophiles (mCPBA, NBS, butyraldehyde) in an attempt to either promote breakdown to the enone (**6p**) or β‐hydroxy ketone (**7p**), or to use **5p** productively for new carbon‐carbon or carbon‐heteroatom bond formation were unsuccessful, leading to degradation or trace amounts of **7p** only.

**Figure 1:**
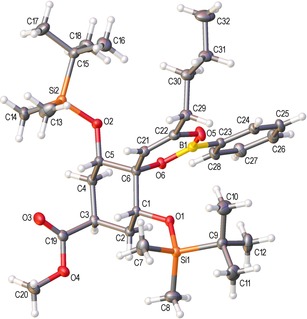
Crystal structure of molecule 1 of **5p** with ellipsoids drawn at the 50 % probability level. The structure contains two crystallographically‐independent molecules of which only one is shown.

The unusual reactivity of propargylic alcohol **3p** is consistent with the observations made during our study of the direct hydration reactions of other substrates (Scheme 4, **3i**–**3j**), in that the presence of the two carbon‐oxygen bonds adjacent to the propargylic alcohol is likely to prevent enone formation *via* destabilisation of the cationic transition state for the elimination of water from intermediate **11**, hydroxy ketone **7** (Scheme 8), or indeed the boron enolate **5p**. Thus, when the propargylic alcohol carbon bears electron‐withdrawing substituents, the Meyer–Schuster rearrangement pathway becomes too high in energy, and protodemetallation of intermediate **8** becomes the only available pathway (Scheme 8), regardless of the protic additive present. With these substrates, the propargylic alcohol itself is also likely to be more acidic than a typical alcohol, and therefore may also be able to promote protodemetallation of the vinylgold intermediate **8**.

In conclusion, we have shown that the gold‐catalysed Meyer–Schuster rearrangement of propargylic alcohols to enones can be diverted to give β‐hydroxy ketone products by the addition of protic additives to the reaction mixture. In combination with the straightforward addition of a terminal alkyne to a carbonyl compound, this offers an interesting alternative to the aldol reaction. Furthermore, we have prepared an unusually stable cyclic boron enolate **5p** through gold‐catalysed reaction of a hindered electron‐deficient propargylic alcohol with a boronic acid. The isolation of **5p** supports our earlier hypothesis3b that cyclic boron enolates **5** are intermediates in the gold‐catalysed reactions of propargylic alcohols using boronic acid additives.

## Experimental Section

Please see the Supporting Information for full details of the experimental procedures, together with ^1^H and ^13^C NMR spectra and crystallographic data for enolate **5p**.^20^

### General Procedure A: Preparation of Propargylic Alcohols

*n‐*Butyllithium (1.6 M in hexanes, 1.2 equiv.) was added dropwise to a stirred solution of alkyne (1 equiv.) in dry THF (1 mL mmol^−1^) at −78 °C under an argon atmosphere. After 30 min aldehyde (1 equiv.) was added and the resulting solution was allowed to warm to room temperature and stirred overnight. The reaction was quenched with saturated aqueous NaHCO_3_ and the organic phase extracted with diethyl ether. The combined organic extracts were washed with brine, dried (MgSO_4_) and concentrated under vacuum. The residue was purified by column chromatography to give the propargylic alcohol.

**Dec‐5‐yne‐4‐ol (3a):^[3]^** yield: 75%; IR (film): *ν*_max_=3331, 2958, 2933, 2873, 2231 cm^−1^; ^1^H NMR (600 MHz, CDCl_3_): *δ*=0.89 (3 H, t, *J*=7.2 Hz), 0.91 (3 H, t, *J*=7.3 Hz), 1.43 (4 H, m), 1.64 (4 H, m), 1.93 (1 H, br s), 2.20 (2 H, td, *J*=7.2, 1.9 Hz), 4.35 (1 H, m); ^13^C NMR (150 MHz, CDCl_3_): *δ*=13.7, 13.9, 18.5, 18.6, 22.0, 30.9, 40.4, 62.6, 81.4, 85.6.

### General Procedure B: Preparation of β‐Hydroxy Ketones using *p*‐Nitrophenol as Additive

[Ph_3_PAuNTf_2_]_2_PhMe (2 mol%) was added to a solution of propargylic alcohol (1 equiv.) and 4‐nitrophenol (1 equiv.) dissolved/suspended in toluene (10 mL g^−1^; sonication was used to dissolve the 4‐nitrophenol as much as possible) and the solution stirred magnetically at room temperature until the starting material had disappeared by TLC (24–48 h). The reaction was quenched with aqueous NH_4_Cl and the organic phase extracted with Et_2_O. The combined organic phases were washed with brine, dried (MgSO_4_), concentrated under vacuum, and the crude product was purified by column chromatography to give the β‐hydroxy ketone.

**7‐Hydroxydecan‐5‐one (7a):^[21]^** yield: 65%; IR (film): *ν*_max_=3429, 2958, 2932, 2873, 1704 cm^−1^; ^1^H NMR (600 MHz, CDCl_3_): *δ*=0.90 (3 H, t, *J*=7.4 HZ), 0.92 (3 H, t, *J*=7.2 Hz), 1.25–1.59 (8 H, m), 2.42 (2 H, t, *J*=7.4 Hz), 2.49 (1 H, dd, *J*=17.6, 8.4 Hz), 2.59 (1 H, dd, *J*=17.6, 2.7 Hz), 4.02–4.06 (1 H, m); ^13^C NMR (150 MHz, CDCl_3_): *δ*=13.9, 14.1, 18.7, 22.3, 25.8, 38.6, 43.5, 49.0, 67.4, 212.7.

## Supporting information

As a service to our authors and readers, this journal provides supporting information supplied by the authors. Such materials are peer reviewed and may be re‐organized for online delivery, but are not copy‐edited or typeset. Technical support issues arising from supporting information (other than missing files) should be addressed to the authors.

Supplementary

## References

[1a] E. B. Bauer , Synthesis 2012, 1131;

[1b] Y. Zhu , L. Sun , P. Lu , Y. Wang , ACS Catal. 2014, 4, 1911.

[2a] R. S. Ramón , N. Marion , S. P. Nolan , Tetrahedron 2009, 65, 1767;

[2b] M. Egi , Y. Yamaguchi , N. Fujiwara , S. Akai , Org. Lett. 2008, 10, 1867; 18393519 10.1021/ol800596c

[2c] R. Ramón , S. Gaillard , A. M. Z. Slawin , A. Porta , A. D‘Alfonso , G. Zanoni , S. P. Nolan , Organometallics 2010, 29, 3665;

[2d] S. I. Lee , J. Y. Baek , S. H. Sim , Y. K. Chung , Synthesis 2007, 2107;

[2e] D. A. Engel , S. S. Lopez , G. B. Dudley , Tetrahedron 2008, 64, 6988;

[2f] D. A. Engel , G. B. Dudley , Org. Lett. 2006, 8, 4027; 16928065 10.1021/ol0616743

[2g] S. S. Lopez , D. A. Engel , G. B. Dudley , Synlett 2007, 949.

[3a] M. N. Pennell , P. G. Turner , T. D. Sheppard , Chem. Eur. J. 2012, 18, 4748; 22374879 10.1002/chem.201102830

[3b] M. N. Pennell , M. G. Unthank , P. Turner , T. D. Sheppard , J. Org. Chem. 2011, 76, 1479. 21265538 10.1021/jo102263t

[4a] L. Ye , L. Zhang , Org. Lett. 2009, 11, 3646; 19637857 10.1021/ol901346k

[4b] T. de Haro , C. Nevado , Chem. Commun. 2011, 47, 248; 10.1039/c002679d 20589279

[4c] M. Yu , G. Zhang , L. Zhang , Org. Lett. 2007, 9, 2147; 17465561 10.1021/ol070637o

[4d] M. Yu , G. Zhang , L. Zhang , Tetrahedron 2009, 65, 1846.

[5a] G. Zhang , Y. Peng , L. Cui , L. Zhang , Angew. Chem. 2009, 121, 3158; 10.1002/anie.200900585 19322869

[5b] B. S. L. Collins , M. G. Suero , M. J. Gaunt , Angew. Chem. 2013, 125, 5911; 10.1002/anie.201301529 23610012

[6] J. M. D′Oyley , A. E. Aliev , T. D. Sheppard , Angew. Chem. 2014, 126, 10923; 10.1002/anie.201405348 PMC4271674 25147077

[7] C. Körner , P. Starkov , T. D. Sheppard , J. Am. Chem. Soc. 2010, 132, 5968. 20380452 10.1021/ja102129c

[8] For regioselective hydration of terminal propargylic alcohols to methyl ketones, see:

[8a] C. Palomo , A. González , J. M. García , C. Landa , M. Oiarbide , S. Rodríguez , A. Linden , Angew. Chem. 1998, 110, 190;

[8b] R. C. Gupta , P. A. Harland , R. J. Stoodley , J. Chem. Soc. Chem. Commun. 1983, 754;

[8c] see also ref. [3a].

[9] For reports of the formation of β-hydroxy ketone by-products during the study of other Au-catalysed reactions of propargylic alcohols, see:

[9a] J.-H. An , H. Yun , S. Shin , S. Shin , Adv. Synth. Catal. 2014, 356, 3749;

[9b] B. Alcaide , P. Almendros , M. T. Quirós , Chem. Eur. J. 2014, 20, 3384. 24532455 10.1002/chem.201304509

[10] For indirect hydration of propargylic alcohols *via* hydrosilylation or hydroboration followed by oxidation, see:

[10a] B. M. Trost , Z. T. Ball , T. Jöge , Angew. Chem. 2003, 115, 3537; 10.1002/anie.200351587 12888974

[10b] B. M. Trost , Z. T. Ball , K. M. Laemmerhold , J. Am. Chem. Soc. 2005, 127, 10028; 16011365 10.1021/ja051578h PMC2581901

[10c] J. K. Park , B. A. Ondrusek , D. T. McQuade , Org. Lett. 2012, 14, 4790. 22946740 10.1021/ol302086v

[11] For Ag-catalysed hydration of propargylic alcohols to α-hydroxy ketones, see: H. He , C. Qi , X. Hu , Y. Guana , H. Jianga , Green Chem. 2014, 16, 3729.

[12] D. Boyall , D. E. Frantz , E. M. Carreira , Org. Lett. 2002, 4, 2605. 12123387 10.1021/ol026282k

[13] The regioselective hydration of alcohol **3a** was carried out in C_6_D_6_ and followed by ^1^H NMR. Rapid initial formation of a small amount of enone **6a** was observed (<5%), followed by slow formation of **7a** and further **6a** over the course of 48 h.

[14] In principle, only a catalytic amount of the protic additive is necessary for the reaction. Hydration of propargylic alcohol **3a** to give **6a**/**7a** proceeded effectively with 0.2 or 0.5 equivalents of PNPOH in NMR experiments. However, inconsistent results were obtained when attempting to carry out preparative scale reactions with catalytic quantities of PNPOH.

[15] Although the hydration reaction requires an equivalent of water to give complete conversion, addition of further water to the reaction mixture was not found to be beneficial, and adventitious moisture appears to be sufficient for good conversions to be obtained. When a hydration reaction of **3a** was conducted under ‘anhydrous conditions’ (pre-drying of PNPOH and PhMe; addition of dried 4 Å molecular sieves to the reaction mixture; reaction conducted under argon atmosphere), a 5:2 ratio of enone **6a**:β-hydroxy ketone **7a** was observed in the ^1^H NMR spectrum of the crude product.

[16] In order to investigate whether a gold phenolate species could be the active catalyst in this reaction, we heated a sample of Ph_3_PAuMe with PNPOH in C_6_D_6_ This led to the disappearance of the methyl signal and the formation of a new species which did not react with propargylic alcohol **3a**. It should be noted, however, that no stable gold phenolate complexes with phosphine ligands have previously been reported. For examples of gold phenolate complexes with NHC ligands, see:

[16a] N. Ibrahim , M. H. Vilhelmsen , M. Pernpointner , F. Rominger , A. S. K. Hashmi , Organometallics 2013, 32, 2576;

[16b] Y. Oonishi , A. Gómez-Suárez , A. R. Martin , S. P. Nolan , Angew. Chem. 2013, 125, 9949; 10.1002/anie.201304182 23897673

[17] K. E. Roth , S. A. Blum , Organometallics 2010, 29, 1712.

[18a] K. Okada , Y. Hosoda , M. Oda , J. Am. Chem. Soc. 1986, 108, 321;

[18b] P. Paetzold , S. Neyses , L. Géret , Z. Anorg. Allg. Chem. 1995, 621, 732.

[19] T. C. Casey , J. Carlisle , P. Tisselli , L. Male , N. Spencer , R. S. Grainger , J. Org. Chem. 2010, 75, 7461. 20936869 10.1021/jo101531b

[20] CCDC 1448535 contains the supplementary crystallographic data for this paper. These data can be obtained free of charge from The Cambridge Crystallographic Data Centre via www.ccdc.cam.ac.uk/data_request/cif.

[21] C. Roche , O. Labeeuw , M. Haddad , T. Ayad , J. P. Genet , V. Ratovelomanana-Vidal , P. Phansavath , Eur. J. Org. Chem. 2009, 3977.

