# Supplementary material for: Intercepting the Gold‐Catalysed Meyer–Schuster Rearrangement by Controlled Protodemetallation: A Regioselective Hydration of Propargylic Alcohols
Source: Adv Synth Catal. 2016 Apr 27;358(9):1519–25. doi: 10.1002/adsc.201600101 (PMC5698882; doi:10.1002/adsc.201600101)
Supplement: Supplementary file 1 — Supplementary [file ADSC-358-1519-s001.pdf]

Advanced Synthesis & Catalysis

Supporting Information

Intercepting the Gold-Catalysed Meyer-Schuster Rearrangement by Controlled Protodemetallation: A Regioselective Hydration of Propargylic Alcohols

Matthew N. Pennell,[a] Michael P. Kyle,[b] Louise Male,[b] Peter G. Turner,[c] Richard S. Grainger,[b]* and Tom D. Sheppard[a]*

[a]*Department of Chemistry, University College London,*
Christopher Ingold Laboratories, 20 Gordon St, London, WC1H 0AJ, UK
[b]*School of Chemistry, University of Birmingham, Edgbaston,*
Birmingham B15 2TT, UK
[c]*GlaxoSmithKline R & D Limited, Medicines Research Centre,*
Gunnels Wood Road, Stevenage, Herts, SG1 2NY, UK

r.s.grainger@bham.ac.uk
tom.sheppard@ucl.ac.uk

Table of Contents

1. Synthesis of Propargylic Alcohols 2
2. Synthesis of β-Hydroxyketones 6
3. Synthesis of Cyclic Boron Enolate **5p** 11
4. ^{1}H and ^{13}C NMR Spectra 13
5. X-Ray Crystallography Data for Enolate **5p** 27
6. References 29

1. Synthesis of Propargylic Alcohols

The preparation of propargylic alcohols **3k**-**3n** and their rearrangement into the corresponding enones **6k**-**6n** was previously described.[1]

General Procedure A

n-Butyllithium (1.6 M in hexanes, 1.2 eq.) was added dropwise to a stirred solution of alkyne (1 eq.) in dry THF (1 mLmmol^{-1}) at −78 ˚C under an argon atmosphere. After 30 min aldehyde (1 eq.) was added and the resulting solution was allowed to warm to rt and stirred overnight. The reaction was quenched with sat. aq. $NaHCO_3$ and the organic phase extracted with diethyl ether. The combined organic extracts were washed with brine, dried ($MgSO_4$) and concentrated *in vacuo*. The residue was purified by column chromatography to give the propargylic alcohol.

Dec-5-yne-4-ol (3a)[1]

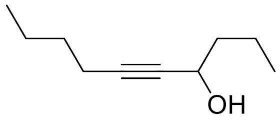

General Procedure A: 3.99 g, 75% yield; vmax (film/cm-1) 3331, 2958, 2933, 2873, 2231; δH (600 MHz, CDCl3) 0.89 (3H, t, *J* 7.2), 0.91 (3H, t, *J* 7.3), 1.43 (4H, m), 1.64 (4H, m), 1.93 (1H, br s), 2.20 (2H, td, *J* 7.2, 1.9), 4.35 (1H, m); δC (150 MHz, CDCl3) 13.7, 13.9, 18.5, 18.6, 22.0, 30.9, 40.4, 62.6, 81.4, 85.6.

1-(Thiophen-3-yl)hex-1-yn-3-ol (3b)[1]

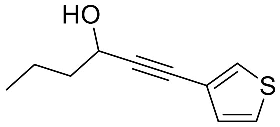

General Procedure A, using EtMgBr (1 eq) instead of BuLi: 900 mg, 46% yield; ν_{max} (film/cm^{-1}) 3353, 2959, 2934, 2872, 2226; δ_H (600 MHz, $CDCl_3$) 0.97 (3H, t, *J* 7.4), 1.53 (2H, app sx, *J* 7.4), 1.72-1.81 (2H, m), 2.07 (1H, br s), 4.58 (1H, t, *J* 6.5), 7.09 (1H, dd, *J* 5.0, 1.1), 7.25 (1H, dd, *J* 5.0, 3.0), 7.42 (1H, dd, *J* 3.0, 1.1); δ_C (150 MHz, $CDCl_3$) 13.9, 18.6, 40.0, 62.9, 80.1, 90.0, 121.8, 125.4, 129.0, 130.0; Found (EI): $[M]^+$ 180.06034, $C_{10}H_{12}OS$ requires 180.06081.

1-(4-Methoxyphenyl)hept-1-yn-3-ol (3c)[1]

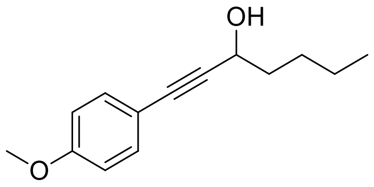

General Procedure A using EtMgBr (1 eq) instead of BuLi: 1.7 g, 78% yield; ν_{max} (film/cm^{-1}) 3383, 2957, 2862, 2839, 2541; δ_H (600 MHz, $CDCl_3$) 0.93 (3H, t, *J* 7.3), 1.38 (2H, app sx, *J* 7.3), 1.46-1.52 (2H, m), 1.74-1.82 (2H, m), 1.84 (1H, br s), 3.80 (3H, s), 4.57 (1H, br t, *J* 6.5), 6.83 (2H, d, *J* 8.8), 7.36 (2H, d, *J* 8.8); δ_C (150 MHz, $CDCl_3$) 14.2, 22.5, 27.5, 37.8, 55.4, 63.1, 84.8, 88.9, 114.0, 114.8, 133.3, 159.7; Found (EI): $[M]^+$ 218.13087, $C_{14}H_{18}O_2$ requires 218.13013.

1-Phenylhex-1-yn-3-ol (3d)[1]

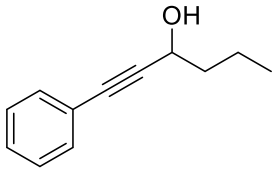

General Procedure A:3.33 g, 96% yield; ν_{max} (ATR/cm^{-1}) 3324, 2958, 2933, 2872; δ_H (500 MHz, $CDCl_3$) 0.99 (3H, t, *J* 7.4), 1.56 (2H, m), 1.47 (2H, m), 4.61 (1H, t, *J* 6.6), 7.31 (3H, m), 7.43 (2H, m); δ_C (150 MHz, $CDCl_3$) 13.9, 18.6, 40.1, 62.9, 84.9, 90.3, 122.8, 128.4, 128.5, 131.8; Found (CI): $[M+H]^+$ 174.1040, $C_{12}H_{15}O$ requires 174.1039; Found (CI): $[M+H]^+$ 174.1040, $C_{12}H_{15}O$ requires 174.1039.

1-(4-(Trifluoromethyl)phenyl)hex-1-yn-3-ol (3e)[1]

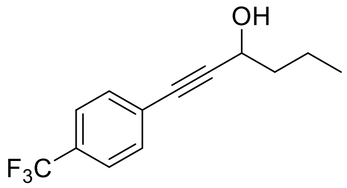

General Procedure A: 2.20g, 91%; ν_{max} (ATR/cm^{-1}) 3314, 2961, 2936, 2875;
δ_H (500 MHz, $CDCl_3$) 0.99 (3H, t, *J* 7.4), 1.55 (2H, m), 1.80 (2H, m), 4.62 (1H, t, *J* 6.6), 7.52 (2H, d, *J* 8.4), 7.56 (2H, d, *J* 8.3); δ_C (600 MHz, $CDCl_3$) 13.8, 18.6, 39.9, 62.8, 83.6, 92.7, 125.2 (q, *J* 3.8), 126.6, 130.1, 130.3, 132.0; Found (EI) $[M]^+$ 242.0915, $C_{13}H_{13}OF_3$ requires 242.0913.

1-(Cyclohex-1-en-1-yl)hex-1-yn-3-ol (3f)[1]

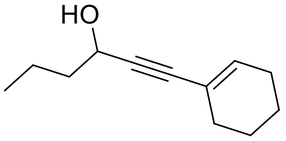

General Procedure A using EtMgBr (1 eq) instead of BuLi: 1.10g , 62% yield; ν_{max} (film/cm^{-1}) 3391, 2940, 2938, 2873, 2187; δ_H (600 MHz, $CDCl_3$) 0.94 (3H, t, *J* 7.4), 1.47 (2H, app sx, *J* 7.4), 1.53-1.74 (6H, m), 2.04-2.12 (5H, m), 4.47 (1H, br t, *J* 6.6), 6.09 (1H, m); δ_C (150 MHz, $CDCl_3$) 13.9, 18.6, 21.6, 22.4, 25.7, 29.3, 40.2, 62.8, 86.7, 87.6, 120.2, 135.3; Found (EI): $[M]^+$ 178.13569, $C_{12}H_{18}O$ requires 178.13522.

1-(4-Methoxyphenyl)-5-phenylpent-1-yn-3-ol (3g)[1]

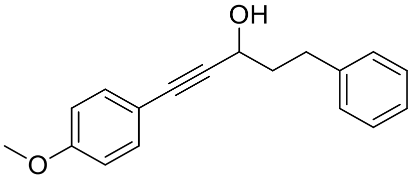

General Procedure A using EtMgBr (1 eq) instead of BuLi: 2.0 g, 75% yield; ν_{max} (film/cm^{-1}) 3364, 3063, 2933, 2876, 2255; δ_H (500 MHz, $CDCl_3$) 2.09-2.16 (2H, m), 2.19 (1H, br s), 2.87 (2H, t, *J* 7.8), 3.81 (3H, s), 4.60 (1H, t, *J* 6.5), 6.85 (2H, d, *J* 8.7), 7.21 (1H, t, *J* 7.5), 7.25 (2H, d, *J* 7.5), 7.31 (2H, t, *J* 7.5), 7.39 (2H, d, *J* 8.7); δ_C (150 MHz, $CDCl_3$) 31.7, 39.5, 55.4, 62.4, 85.3, 88.6, 114.1, 114.8, 126.1, 128.6, 128.7, 133.3, 141.5, 159.8; Found (EI): $[M]^+$ 266.12974, $C_{18}H_{18}O_2$ requires 266.13013.

1-Cyclopentyl-5-methylhex-1-yn-3-ol (3h)

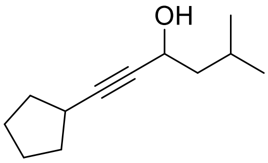

General Procedure A: 1.68 mmol, 78%; ν_{max} (film/cm^{-1}) 3317, 2965, 2870, 2230; δ_H (600 MHz, $CDCl_3$) 0.90 (3H, d, *J* 6.7), 0.92 (3H, d, *J* 6.7), 1.46-1.61 (6H, m), 1.67-1.72 (2H, m), 1.76-1.84 (2H, m), 1.85-1.92 (2H, m), 2.57-2.63 (1H, m), 4.38 (1H, br t, *J* 7.0); δ_C (150 MHz, $CDCl_3$) 22.6, 22.7, 24.9, 25.0, 30.2, 33.91, 33.93, 47.4, 61.3, 81.1, 89.7; Found (CI): $[M-OH]^+$ 163.14810, $C_{12}H_{19}$ requires 163.14868.

1,1,1-Trifluoro-2-phenyl-oct-3-yn-2-ol (3i)[2]

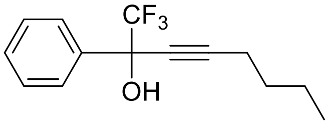

General Procedure A: 9.44g, 85% yield; ν_{max} (film/cm^{-1}) 3464, 2961, 2936, 2875, 2245; δ_H (500 MHz, $CDCl_3$) 0.97 (3H, t, *J* 7.3), 1.43-1.52 (2H, m), 1.56-1.53 (2H, m), 2.35 (2H, t, *J* 7.0), 3.3 (1H, s), 7.42 (3H, m), 7.78 (2H, m); δ_C (125 MHz, $CDCl_3$) 13.6, 18.4, 22.0, 30.2, 73.0 (q, *J* 32.4), 78.4, 89.7, 123.5 (q, *J* 286.1), 127.3, 128.1, 129.3, 135.9.

1,1,1-Trifluoro-2-methyl-oct-3-yn-2-ol (3j)

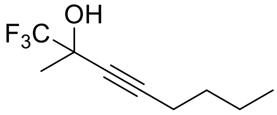

General Procedure A: 6.13 g, 87% yield; ν_{max} (film/cm^{-1}) 3408, 2962, 2938, 2877, 2256; δ_H (600 MHz, $CDCl_3$) 0.90 (3H, t, *J* 7.3), 1.35-1.42 (2H, app sx, *J* 7.3), 1.46-1.51 (2H, qn, *J* 7.3), 1.58 (3H, s), 2.21 (2H, t, *J* 7.3), 2.86 (1H, s); δ_C (150 MHz, $CDCl_3$) 13.5, 18.2, 21.9, 23.3, 30.2, 68.6 (q, *J* 32.5), 76.5, 87.6, 124.1 (q, *J* 282.7); Found (CI): $[M+H]^+$ 177.08874, $C_9H_{12}F_3$ requires 177.08911.

4-Methyl-1-phenylpent-1-yn-3-ol ((*R*)-3o)[3]

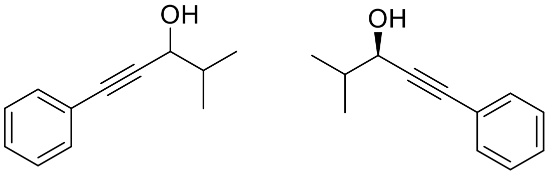

Racemic sample prepared according to General procedure A (1.53 g, 88% yield); Enantioenriched sample prepared accourding to a literature procedure[3] (70 mg, 80% yield, 95:5 er; Daicel Chiralpack AD, hexane:^{i}PrOH 80:20, flow rate 1 mLmin^{-1}. t_R(*S*)=9.5 min, t_R(*R*)=10.1 min); ν_{max} (film/cm^{-1}) 3351, 2961, 2928, 2872, 2219; δ_H (600 MHz, $CDCl_3$) 1.06 (3H, d, *J* 6.8), 1.08 (3H, d, *J* 6.8), 1.96-2.01 (1H, m), 2.25 (1H, br s), 4.41 (1H, d, *J* 5.7), 7.28-7.32 (3H, m), 7.43-7.45 (2H, m); δ_C (150 MHz, $CDCl_3$) 17.7, 18.4, 32.8, 68.5, 85.7, 89.1, 122.9, 128.4, 128.5, 131.8; Found (EI): $[M]^+$ 174.10447, $C_{12}H_{14}O$ requires 174.10391. $[\alpha]_D^{22}$ –0.010 (*c* 1 in $CHCl_3$).

2. Preparation of β-hydroxyketones

General Procedure B

$[Ph_3PAuNTf_2]_2PhMe$ (2 mol%) was added to a solution of propargylic alcohol (1 eq.) and 4-Nitrophenol (1 eq.) dissolved/suspended in toluene (10 mL/g; sonication was used to dissolve the 4-nitrophenol as much as possible) and the solution stirred magnetically at room temperature until starting material had disappeared by TLC (24-48 h). The reaction was quenched with aq. NH_4Cl and the organic phase extracted with Et_2O. The combined organic phases were washed with brine, dried ($MgSO_4$), concentrated *in vacuo*, and the crude product was purified by column chromatography to give β-hydroxyketone.

General Procedure C

$[Ph_3PAuNTf_2]_2PhMe$ (2 mol%) was added to a solution of propargylic alcohol (1 eq.) and boric acid (0.2 eq.) dissolved/suspended in toluene (5 mL/g) and the solution stirred magnetically at 80 °C overnight until starting material had disappeared (TLC). The reaction was concentrated *in vacuo*, and the crude product was purified by column chromatography to give the β-hydroxyketone.

7-Hydroxydecan-5-one (7a)[4]

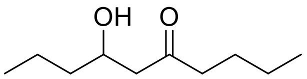

General procedure B; 65% yield; ν_{max} (film/cm^{-1}) 3429, 2958, 2932, 2873, 1704; δ_H (600 MHz, $CDCl_3$) 0.90 (3H, t, *J* 7.4), 0.92 (3H, t, *J* 7.2), 1.25-1.59 (8H, m), 2.42 (2H, t, *J* 7.4), 2.49 (1H, dd, *J* 17.6, 8.4), 2.59 (1H, dd, *J* 17.6, 2.7), 4.02-4.06 (1H, m); δ_C (150 MHz, $CDCl_3$) 13.9, 14.1, 18.7, 22.3, 25.8, 38.6, 43.5, 49.0, 67.4, 212.7. Data in agreement with the literature.[4]

3-Hydroxy-1-(thiophen-3-yl)hexan-1-one (7b)

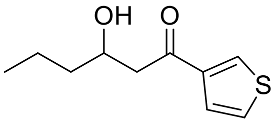

General procedure B; 62% yield; ν_{max} (film/cm^{-1}) 3505, 2962, 2933, 2875, 1667; δ_H (600 MHz, $CDCl_3$) 0.95 (3H, t, *J* 7.3), 1.36-1.63 (4H, m), 2.95 (1H, dd, *J* 17.4, 9.1), 3.08 (1H, dd, *J* 17.4, 2.6), 3.24 (1H, br s) 4.19-4.23 (1H, m), 7.33 (1H, dd, *J* 5.2, 2.8),

7.54 (1H, dd, *J* 5.2, 1.2), 8.07 (1H, dd, *J* 2.8, 1.2); δ_C (150 MHz, $CDCl_3$) 14.2, 18.9, 38.8, 46.3, 67.7, 126.7, 126.8, 132.7, 142.3, 195.4; Found (CI): $[M+H]^+$ 199.07955, $C_{10}H_{15}O_2S$ requires 199.07928.

3-Hydroxy-1-(4-methoxyphenyl)heptan-1-one (7c)

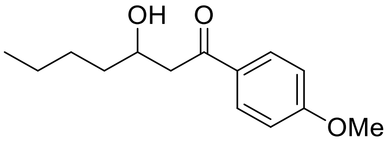

General Procedure B; 75% yield; ν_{max} (film/cm^{-1}) 3502, 3003, 2958, 2933, 1713; δ_H (600 MHz, $CDCl_3$) 0.92 (3H, t, *J* 7.3), 1.33-1.66 (6H, m), 2.97 (1H, dd, *J* 17.5, 9.2), 3.14 (1H, dd, *J* 17.5, 2.4), 3.39 (1H, s), 3.88 (3H, s), 4.16-4.22 (1H, m), 6.94 (2H, d, *J* 8.8), 7.95 (2H, d, *J* 8.8); δ_C (150 MHz, $CDCl_3$) 14.1, 22.7, 27.8, 36.3, 44.6, 55.6, 68.0, 113.9, 130.0, 130.5, 163.9, 199.7; Found (EI): $[M+H]^+$ 249.14714, $C_{14}H_{21}O_3$ requires 249.14852.

3-Hydroxy-1-phenylhexan-1-one (7d)

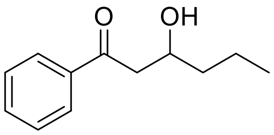

General Procedure B; 42% yield; ν_{max} (ATR/cm^{-1}) 3537, 3063, 2952, 2930, 1670; δ_H (500 MHz, $CDCl_3$) 0.96 (3H, t, *J* 7.1), 1.38-1.67 (4H, m), 3.05 (1H, dd, *J* 17.7, 9.1), 3.17 (1H, dd, *J* 17.7, 2.6), 3.23 (1H, d, *J* 3.1), 4.18-4.28 (1H, m), 7.48 (2H, m), 7.56-7.61 (1H, m), 7.95 (2H, m); δ_C (125 MHz, $CDCl_3$) 14.1, 18.9, 38.7, 45.1, 67.6, 128.1, 128.8, 133.6, 136.9, 201.1; Found (CI): $[M+H]^+$ 193.1224, $C_{12}H_{17}O_2$ requires 193.1223.

3-Hydroxy-1-(4-(trifluoromethyl)phenyl)hexan-1-one (7e)

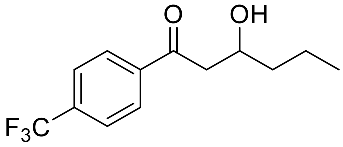

General Procedure B; 20% yield; ν_{max} (ATR/cm^{-1}) 3349, 2957, 2926, 2876, 1668; δ_H (600 MHz, CDCl3) 0.97 (3H, t, *J* 7.2), 1.41-1.66 (4H, m), 3.10 (1H, dd, *J* 17.7, 8.8), 3.17 (1H, dd, *J* 17.7, 2.8), 4.23-4.30 (1H, m), 7.75 (2H, d, *J* 8.2), 8.07 (2H, d, *J* 8.2); δ_C (MHz, CDCl3) 14.1, 18.9, 38.8, 45.6, 67.5, 123.6 (q, *J* 272.8),125.9 (q, *J* 3.7),

128.6, 134.8 (q, *J* 32.8), 139.5, 200.0; Found (CI): $[M+H]^+$ 260.1020, $C_{13}H_{16}F_3O_2$ requires 260.1019.

3-Hydroxy-1-(4-(trifluoromethyl)phenyl)hexan-2-one

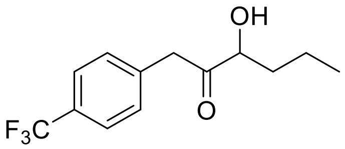

Obtained as a byproduct during the preparation of **7e**; 6% yield; ν_{max} (ATR) 3447, 2962, 2935, 2875, 1715; δ_H (600 MHz, $CDCl_3$) 0.97 (3H, t, *J* 7.3), 1.40 (1H, m), 1.51 (1H, m), 1.60 (1H, m), 1.87 (1H, m), 3.27 (1H, br s), 3.84 (1H, d, *J* 16.2), 3.89 (1H, d, *J* 16.2), 4.31 (1H, dd, *J* 7.7, 3.6), 7.33 (2H, d, *J* 8.0), 7.61 (2H, d, *J* 8.0); δ_C (150 MHz, $CDCl_3$) 14.0, 18.3, 35.8, 44.5, 76.4, 124.16 (q, *J* 272), 125.8, 129.8 (q, *J* 32.6), 130.0, 137.1, 209.1; Found (EI): $[M+H]^+$ 260.1021, $C_{13}H_{16}F_3O_2$ requires 260.1019.

1-(Cyclohex-1-en-1-yl)-3-hydroxyhexan-1-one (7f)

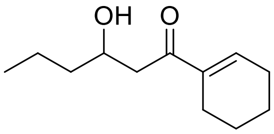

General procedure B; 69% yield; ν_{max} (film/cm^{-1}) 3502, 2961, 2935, 2875, 1710; δ_H (600 MHz, $CDCl_3$) 0.93 (3H, t, *J* 7.3), 1.35-1.42 (2H, m), 1.46-1.54 (2H, m), 1.59-1.67 (4H, m), 2.21-2.28 (4H, m), 2.66 (1H, dd, *J* 17.3, 9.4), 2.85 (1H, dd, *J* 17.3, 2.4), 3.34 (1H, br s), 4.04-4.09 (1H, br m), 6.92-6.95 (1H, m); δ_C (150 MHz, $CDCl_3$) 14.2, 18.9, 21.6, 21.9, 23.0, 26.3, 38.8, 43.3, 67.8, 139.5, 141.5, 202.3; Found (CI): $[M+H]^+$ 197.15389, $C_{12}H_{21}O_2$ requires 197.15415.

3-Hydroxy-1-(4-methoxyphenyl)-5-phenylpentan-1-one (7g)[5]

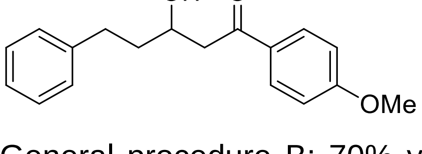

General procedure B; 70% yield; ν_{max} (film/cm^{-1}) 3542, 3054, 2987, 1712; δ_H (600 MHz, $CDCl_3$) 1.76-1.83 (1H, m, $CHCH_2CH_2$), 1.90-1.97 (1H, m, $CHCH_2CH_2$), 2.72-2.78 (1H, m, $CHCH_2CH_2$), 2.86-2.92 (1H, m, $CHCH_2CH_2$), 3.00 (1H, dd, *J* 17.5, 9.2, $OCCH_2$), 3.12 (1H, dd, *J* 17.5, 2.4, $OCCH_2$), 3.50 (1H, br d, *J* 2.78, OH), 3.87 (3H, s, OMe), 4.19-4.24 (1H, br m, C*H*OH), 6.93 (2H, d, *J* 8.9, Ar-H), 7.19 (1H, t, *J* 7.2, Ar-

H), 7.23 (2H, d, *J* 7.0, Ar-H), 7.29 (2H, t, *J* 7.6, Ar-H), 7.92 (2H, d, *J* 8.9, Ar-H); δ_C (150 MHz, $CDCl_3$) 32.0, 38.3, 44.6, 55.7, 67.3, 113.9, 126.0, 128.5, 128.6, 129.9, 130.5, 142.1, 164.0, 199.6; Found (EI): $[M+Na]^+$ 307.1306, $C_{18}H_{20}O_3Na$ requires 307.1310.

1-Cyclopentyl-3-hydroxy-5-methylhexan-1-one (7h)

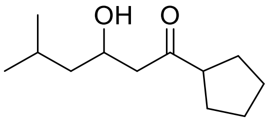

General Procedure B; 50% yield; ν_{max} (film/cm^{-1}) 3480, 2980, 2945, 2898, 1700; δ_H (600 MHz, $CDCl_3$) 0.91 (6H, dd, *J* 6.8, 2.3), 1.10-1.15 (1H, m), 1.44-1.49 (1H, m), 1.57-1.84 (9H, m), 2.52 (1H, dd, *J* 17.7, 9.0), 2.62 (1H, dd, *J* 17.7, 2.6), 2.85 (1H, qn, *J* 7.9), 3.13 (1H, br s), 4.11 (1H, br m); δ_C (150 MHz, $CDCl_3$) 22.1, 23.5, 24.5, 26.1, 28.8, 28.9, 45.6, 48.5, 52.1, 65.9, 215.0; Found (CI): $[M+H]^+$ 199.17059, $C_{12}H_{23}O_2$ requires 199.16980.

1,1,1-Trifluoro-2-hydroxy-2-phenyl-octan-4-one (7i)

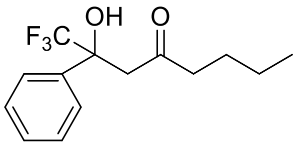

General procedure C; 80% yield; ν_{max} (film/cm^{-1}) 3395, 2961, 2935, 2875, 1702; δ_H (600 MHz, $CDCl_3$) 0.85 (3H, t, *J* 7.4), 1.21 (2H, sx, *J* 7.4), 1.48 (2H, qn, *J* 7.4), 2.39 (1H, dt, *J* 17.3, 7.3), 2.50 (1H, dt, *J* 17.3, 7.3), 3.16 (1H, d, *J* 17.3), 3.32 (1H, d, *J* 17.3), 5.62 (1H, s), 7.34-7.42 (3H, m), 7.56 (2H, d, *J* 7.7); δ_C (150 MHz, $CDCl_3$) 13.8, 22.1, 25.2, 44.4, 44.8, 76.2 (q, *J* 29.3), 124.6 (q, *J* 282.5), 126.3, 128.6, 128.9, 137.6, 211.7; Found (CI): $[M+H]^+$ 275.12623, $C_{14}H_{18}O_2F_3$ requires 275.12589.

1,1,1-Trifluoro-2-hydroxy-2-methyl-octan-4-one (7j)

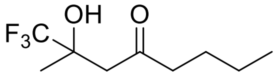

General Procedure C; 90% yield; ν_{max} (film/cm^{-1}) 3446, 2960, 2930, 2873, 1725; δ_H (600 MHz, $CDCl_3$) 0.91 (3H, t, *J* 7.5), 1.32 (2H, sx, *J* 7.5), 1.39 (3H, s), 1.53-1.59 (2H, m), 2.47-2.50 (2H, m), 2.55 (1H, d, *J* 16.8), 2.92 (1H, d, *J* 16.8), 5.2 (1H, s); δ_C

(150 MHz, $CDCl_3$) 13.9, 22.2, 25.3, 44.4, 44.6, 73.2 (q, *J* 29.0), 125.8 (q, *J* 287.4), 212.0; Found (CI): $[M+H]^+$ 213.11064, $C_9H_{16}O_2F_3$ requires 213.11024.

3-Hydroxy-4-methyl-1-phenylpentan-1-one ((*S*)-7o)[6]

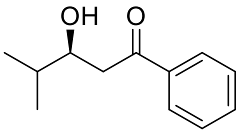

General Proecedure B; 65% yield, 95:5 er (Daicel Chiralpack AD, hexane:^{i}PrOH 80:20, flow rate 1 mLmin^{-1}. t_R(*S*)=12.8 min, t_R(*R*)=14.1 min); ν_{max} (film/cm^{-1}) 3472, 2960, 2928, 2874, 1676; δ_H (600 MHz, $CDCl_3$) 0.99 (3H, d, *J* 6.8), 1.02 (3H, d, *J* 6.8), 1.77-1.83 (2H, m), 3.03 (1H, dd, *J* 17.4, 9.5), 3.17 (1H, dd, *J* 17.4, 2.2), 3.19 (1H, br s), 3.98-4.01 (1H, m), 7.45 (2H, t, *J* 7.6), 7.59 (1H, t, *J* 7.6), 7.95-7.98 (2H, m); δ_C (150 MHz, $CDCl_3$) 18.0, 18.7, 33.2, 42.0, 72.5, 128.2, 128.8, 133.6, 137.0, 201.5; Found (EI): $[M]^+$ 193.12302, $C_{12}H_{17}O_2$ requires 193.12285.

3. Preparation of Cyclic Boron Enolate 5p

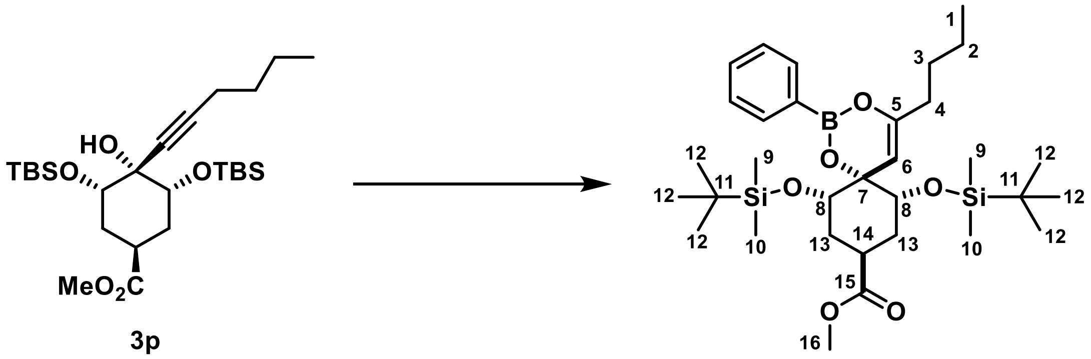

3p **5p**

To a solution of propargylic alcohol **3p** (349 mg, 0.7 mmol) in toluene (0.7 mL) was added phenylboronic acid (85 mg, 0.7 mmol) and PPh_3AuNTf_2 (5 mol%). The reaction was left to stir overnight at rt. The crude mixture had the solvent removed under reduced pressure and the remaining residue purified directly by column chromatography (9:1 petroleum ether: diethyl ether) to give compound **5p** as a white solid (268 mg, 64%). M.p. 33-35 ^oC. R_f: 0.69 (9:1 petroleum ether: diethyl ether) visualised in vanillin; δ_H (400 MHz, C_6D_6) 8.32 – 8.24 (2 H, m, Ar-H), 7.41 – 7.33 (3 H, m, Ar-H), 4.44 (1 H, s, H6), 3.77 (2 H, dd, *J* 9.9, 6.1, H8), 3.46 (3 H, s, H16), 2.73 – 2.51 (1 H, m, H14), 2.36 (2 H, t, *J* 7.3, H4), 2.33 – 2.21 (4 H, m, H13 ax & eq), 1.79 (2 H, p, *J* 7.3, H3), 1.49 (2 H, h, *J* 7.3, H2), 1.05 (3 H, t, *J* 7.3, H1), 1.01 (18 H, s, H12), 0.23 (6 H, s, H9), 0.21 (6 H, s, H10); δ_C (101 MHz, C_6D_6) 174.8 (C, C15), 153.1 (C, C5), 134.7 (Ar-C), 131.2 (Ar-C), 128.2 (Ar-C), 127.9 (Ar-C), 103.1 (CH, C6), 80.2 (C, C7), 72.5 (CH, C8), 51.3 (CH_3, C16), 36.9 (CH, C14), 34.4 (CH_2, C4), 30.3 (CH_2, C13), 28.8 (CH_2, C3), 25.9 (CH_3, C12), 23.1 (CH_2, C2), 18.2 (C, C11), 14.2 (CH_3, C1), -4.0 (CH_3, C9) , -4.51 (CH_3, C10); ^{11}B NMR δ (128 MHz, C_6D_6) 10.8; MS ES^+ HRMS (ES): *m*/*z*(%) = 603.3713 [M + H] (calcd for $C_{32}H_{56}O_6Si_2{}^{11}B$: 603.3708), 603.4- 100% [$M^{11}B$ + H], 602.4-20% [$M^{10}B$ + H]; ν_{max} 2929, 2954, 2857, 2895 (CH), 1734 (C=O Ester), 1696 (C=C enol boronate stretch).

Hydrolysis of cyclic boron enolate 5p to β-hydroxyketone 7p.

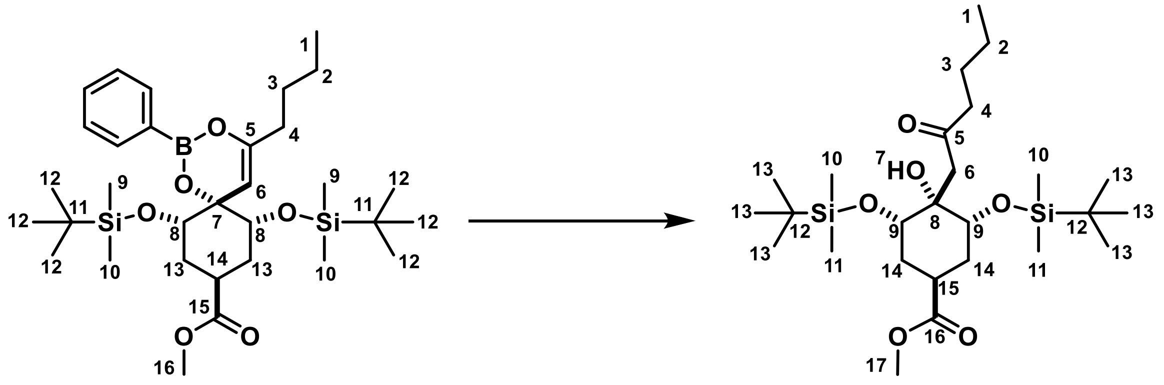

5p **7p**

To a solution of enol boronate **5p** (44 mg, 0.07 mmol) in toluene (0.07 mL) was added H_2O (1 equiv). TsOH.H_2O (1 equiv.) was added and the reaction set to reflux overnight. After cooling to room temperature, the crude reaction mixture was subjected to column chromatography, eluting with 9:1 petroleum ether: diethyl ether, to give β-hydroxyketone **7p** as a colourless oil (16 mg, 42%); δ_H (400 MHz, C_6D_6) 3.98 (2 H, dd, *J* 9.8, 4.7, H9), 3.73 (3 H, s, H17), 2.77 (1 H, tt, *J* 9.8, 4.7, H15), 2.73 (2 H, s, H6), 2.40 (2 H, t, *J* 7.5, H4), 2.00 (2 H, dt, *J* 13.2, 4.7, H14 eq), 1.90 (2 H, ddd, *J* 13.2, 9.8, 4.7, H14 ax), 1.57 (1 H, bs, H7), 1.49 (2 H, p, *J* 7.3, H3), 1.29 (2 H, h, *J* 7.3, H2), 0.93 – 0.85 (21 H, m, H1, H13), 0.08 (6 H, s, H10), 0.01 (6 H, s, H11).; δ_C (101 MHz, C_6D_6) 209.8 (C, C5), 175.2 (C, C16), 75.3 (C, C8), 70.8 (CH, C9), 52.0 (CH_3, C17), 46.0 (CH_2, C6), 44.0 (CH_2, C4), 35.5 (CH, C15), 30.9 (CH_2, C14), 26.1 (CH_3, C13), 25.7 (CH_2, C3), 22.4 (C, C12), 18.2 (CH_2, C2), 14.0 (CH_3, C1), -4.2 (C10), -4.7 (C11); MS ES^+ HRMS (ES): *m*/*z*(%) = 517.3373 [M + H] (calcd for $C_{26}H_{53}O_6Si_2$: 517.3381), 367.2- 100% [M - OH - OTBS - H], 517.3- 80% [M + H]; ν_{max} 3554 (OH), 2954, 2930, 2857, 2895 (CH), 1735 (CO_2Me), 1713 (C=O).

4. ¹H and ¹³C NMR Spectra

7-Hydroxydecan-5-one (7a)

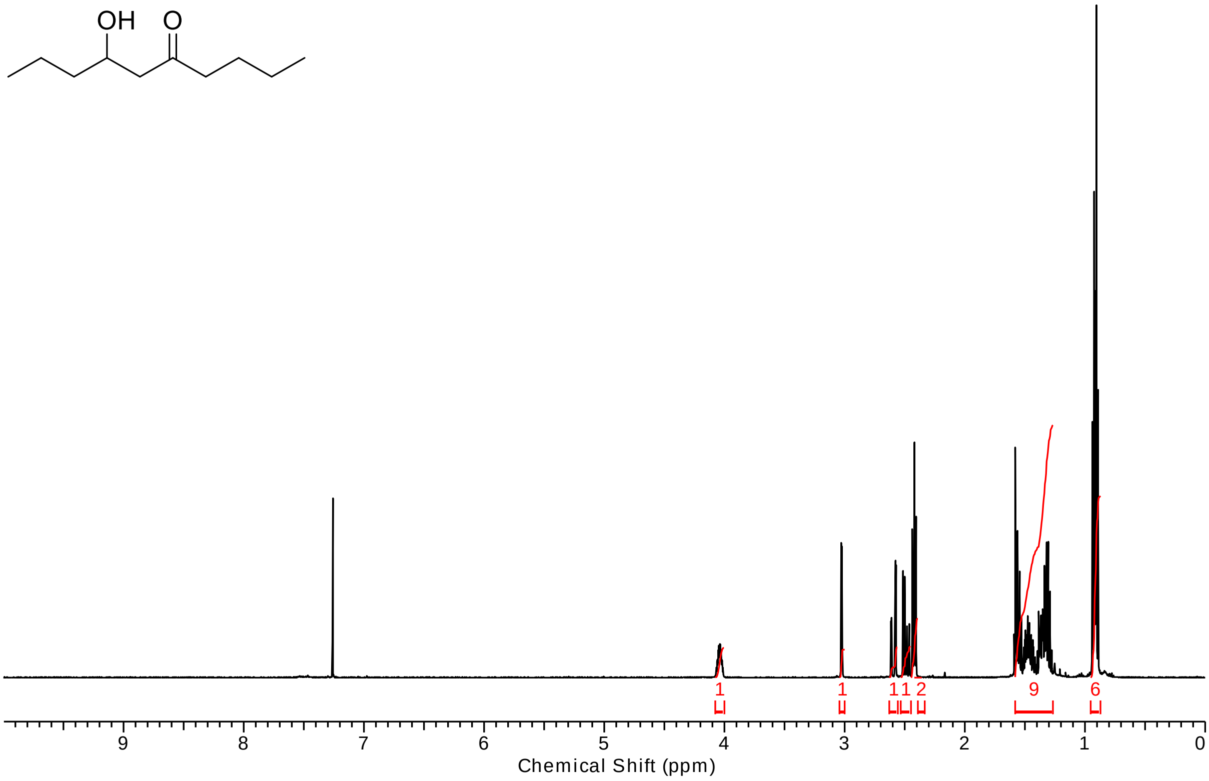

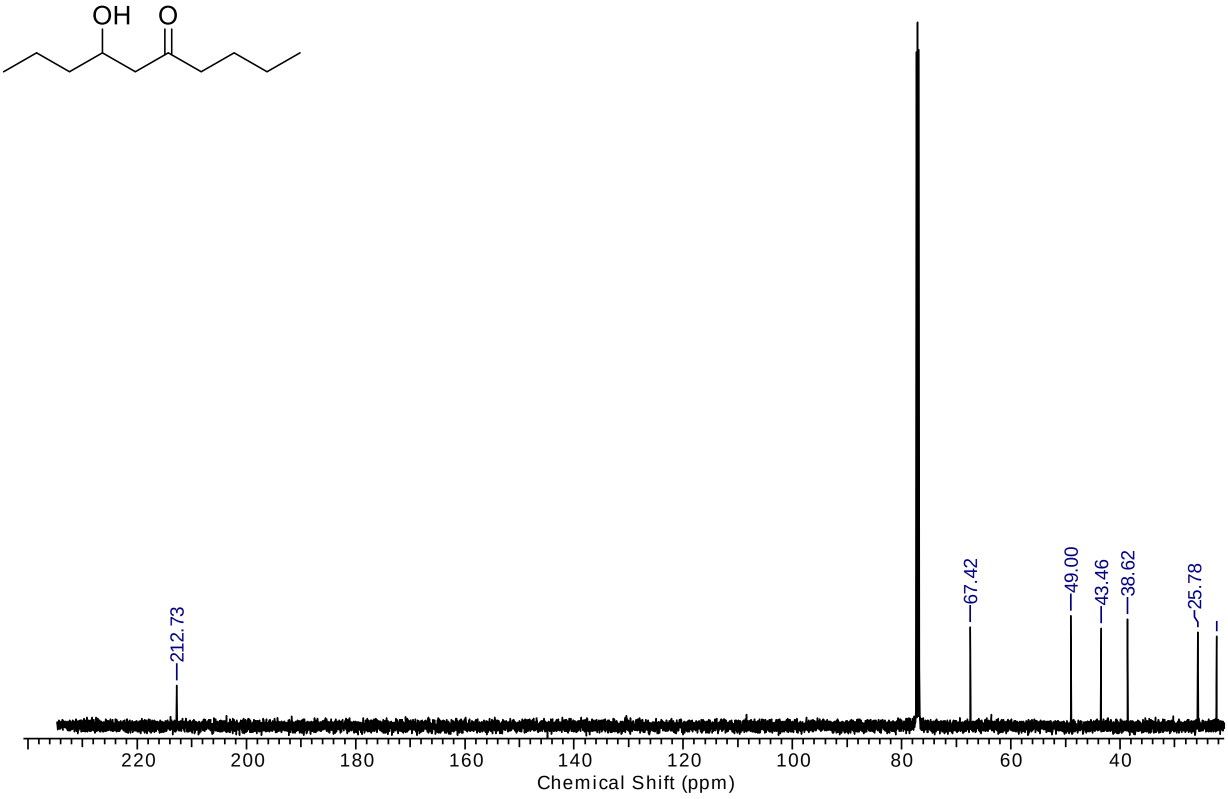

3-Hydroxy-1-(thiophen-3-yl)hexan-1-one (7b)

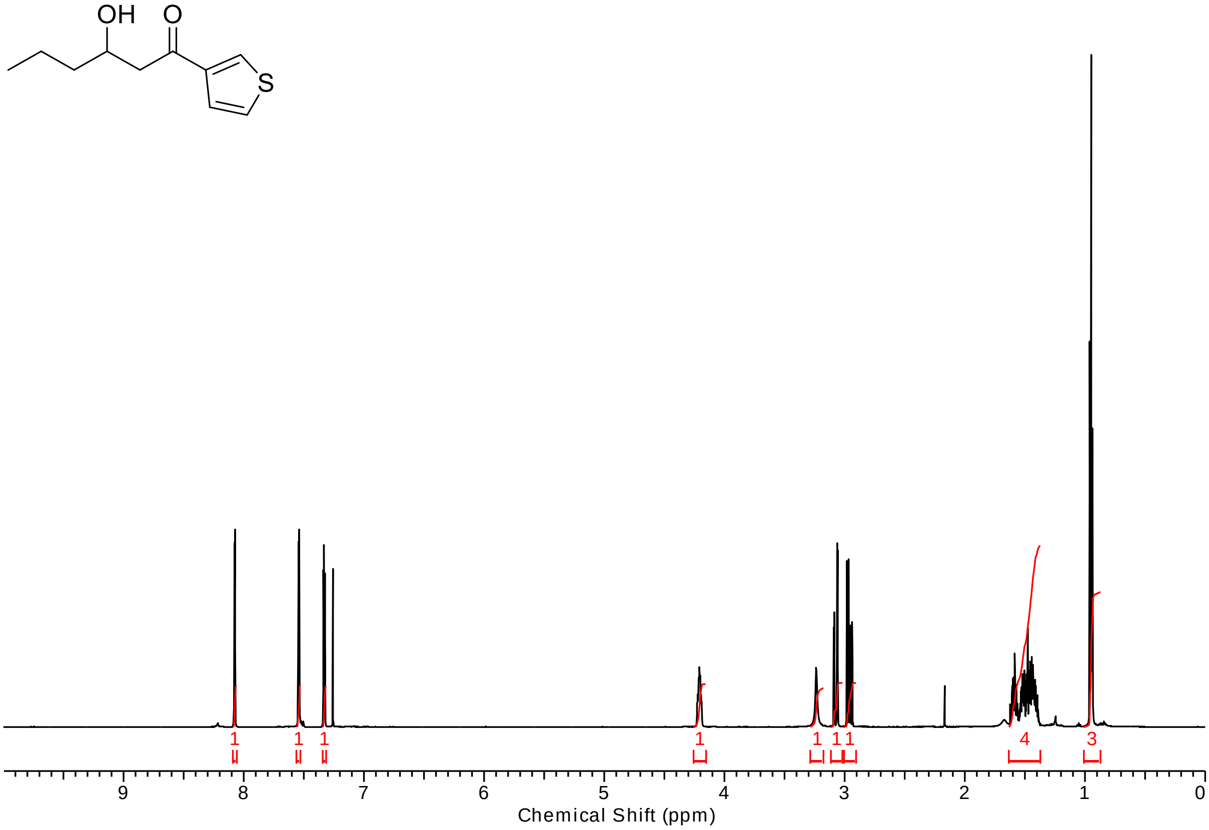

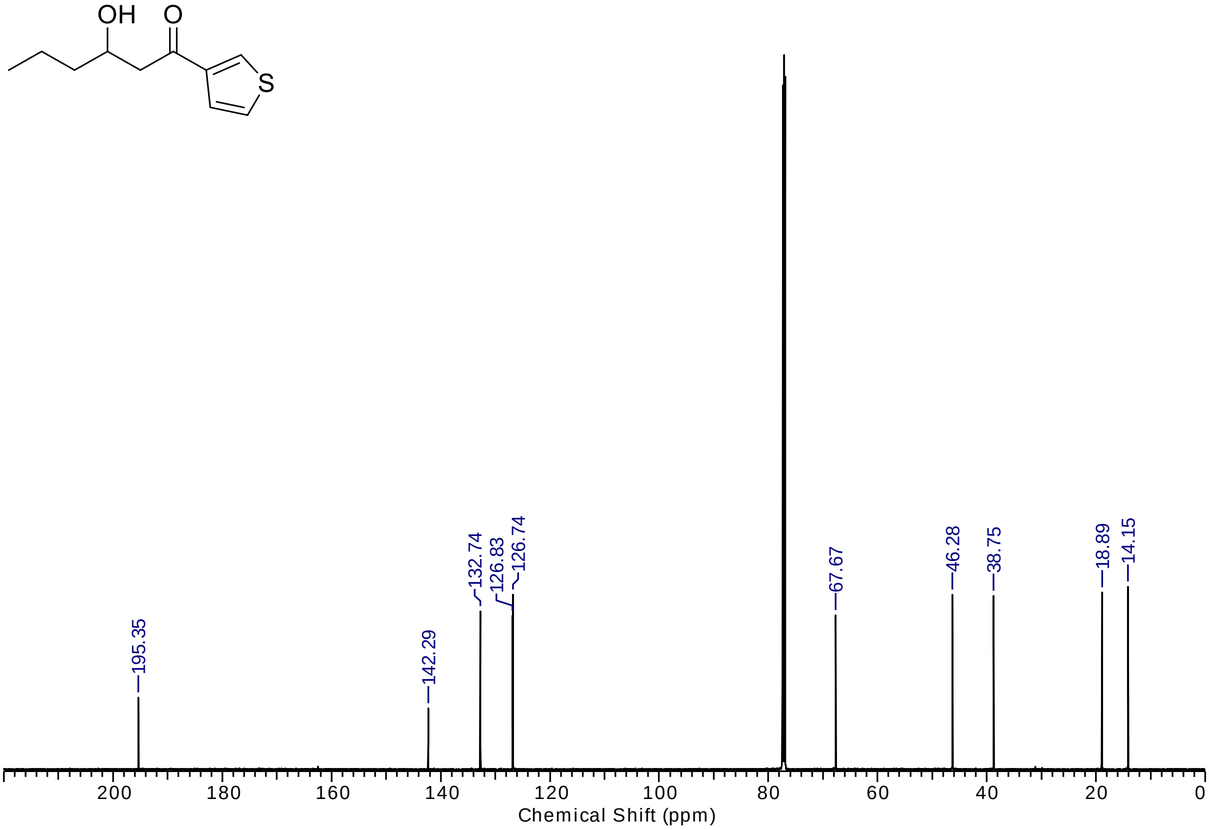

3-Hydroxy-1-(4-methoxyphenyl)heptan-1-one (7c)

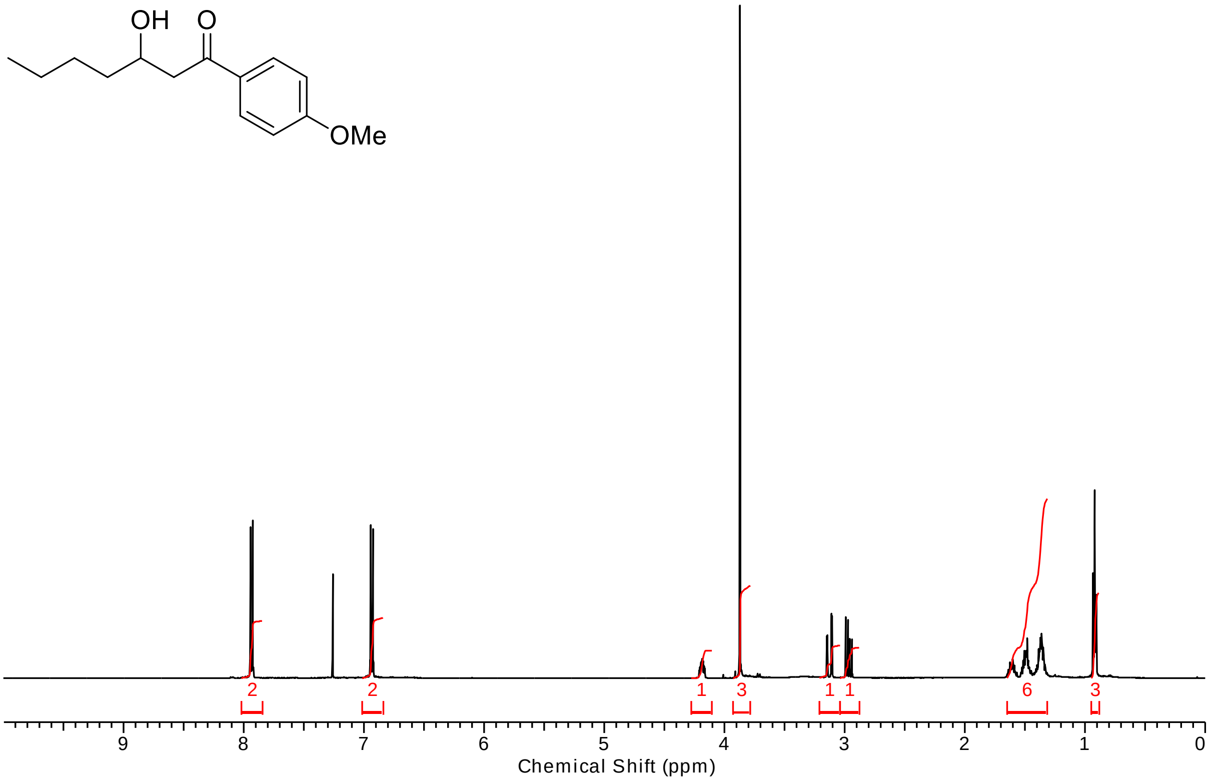

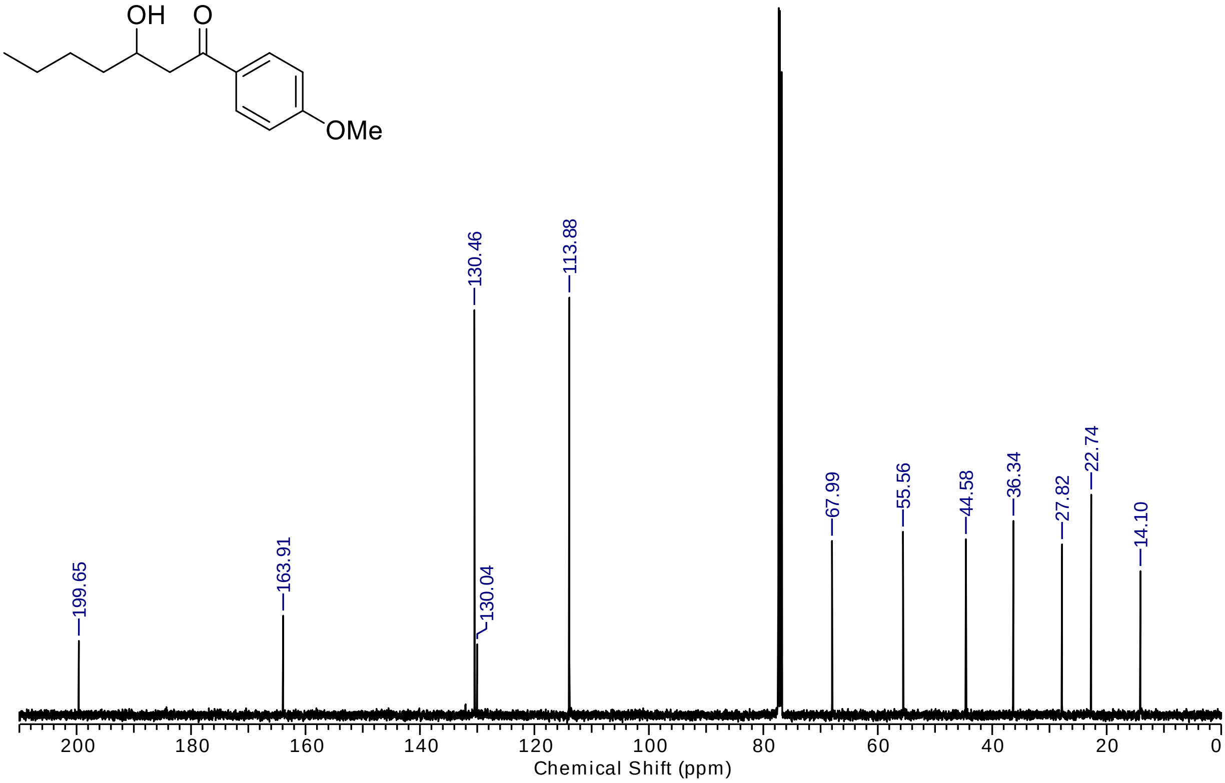

3-Hydroxy-1-phenylhexan-1-one (7d)

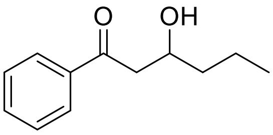

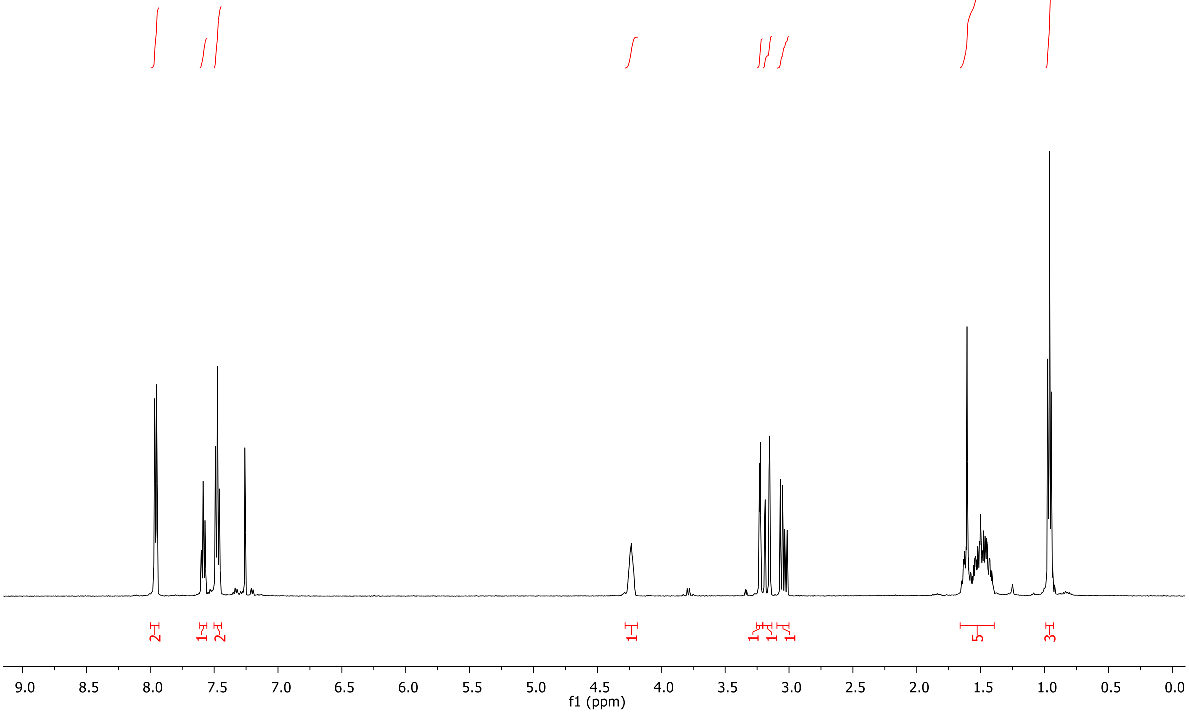

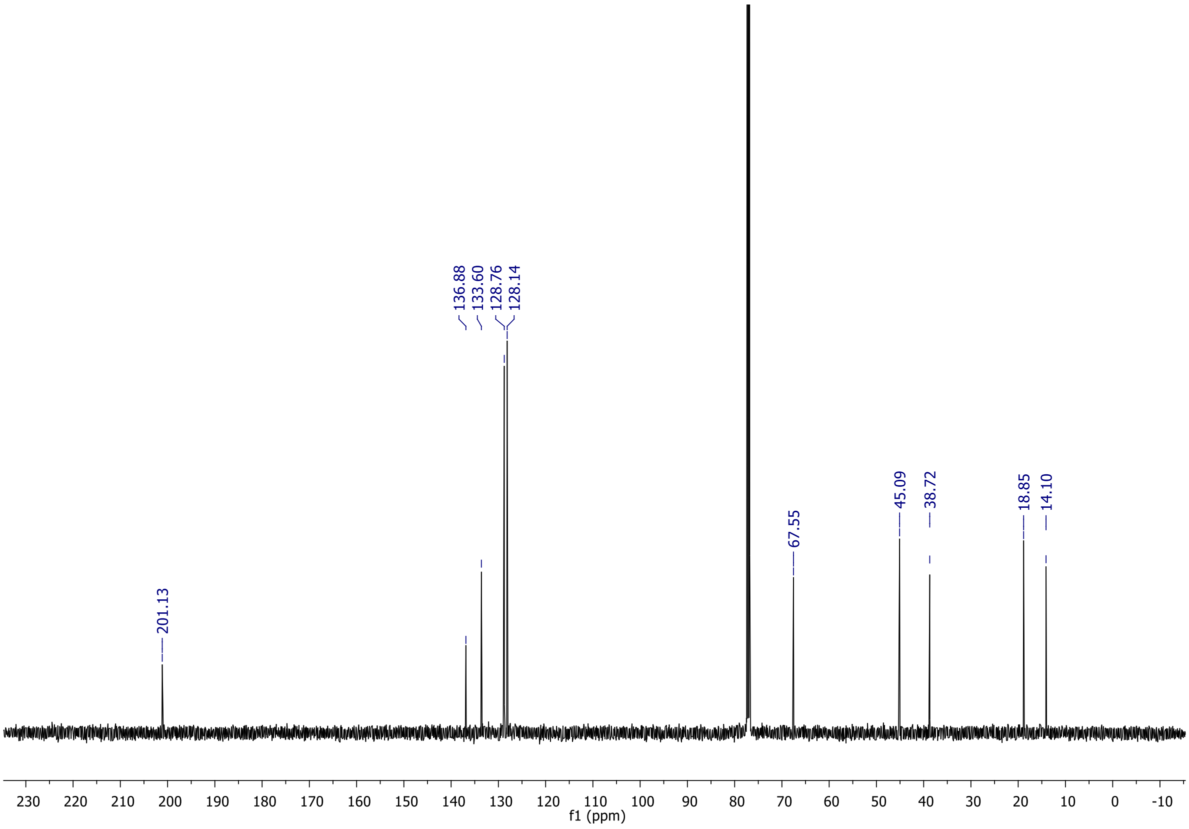

3-Hydroxy-1-(4-(trifluoromethyl)phenyl)hexan-1-one (7e)

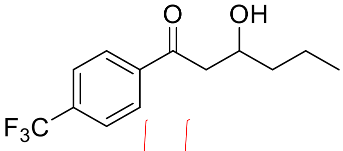

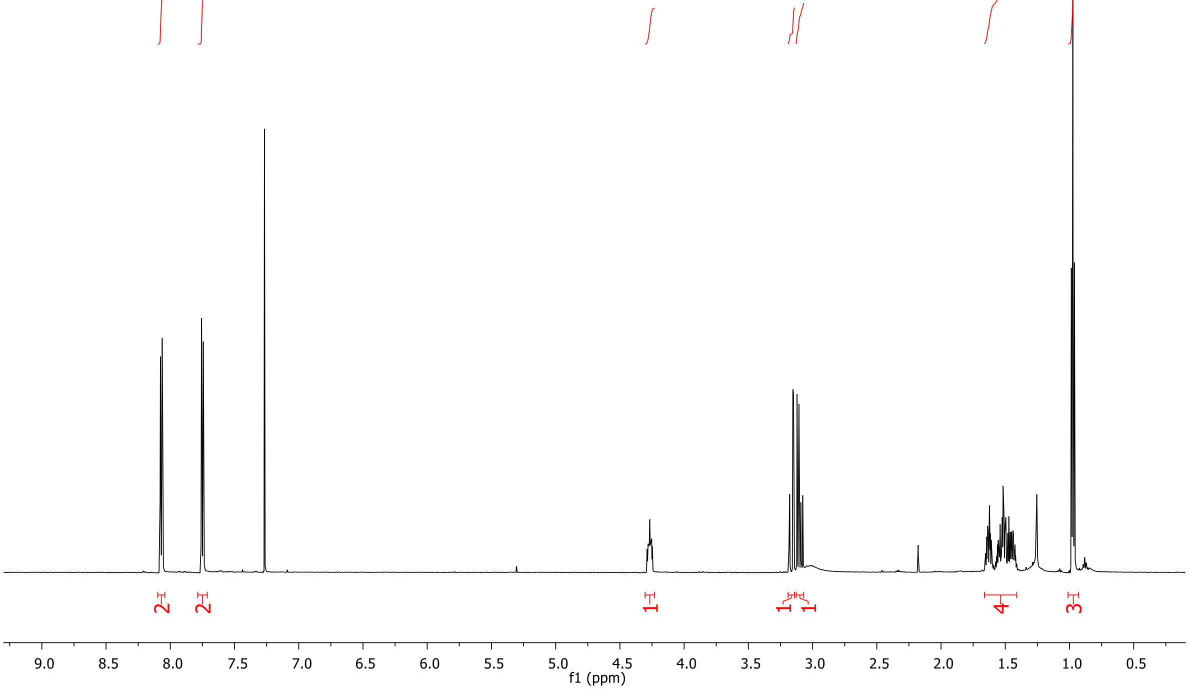

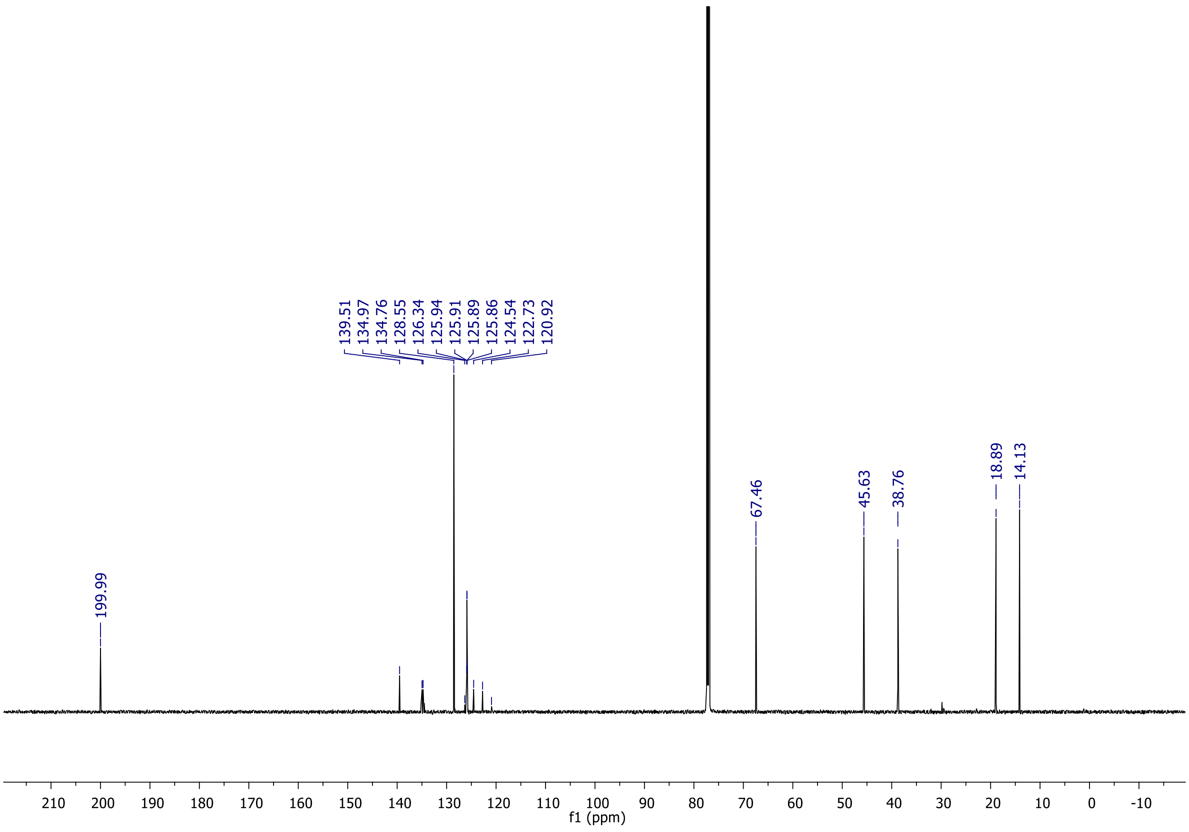

1-(Cyclohex-1-en-1-yl)-3-hydroxyhexan-1-one (7f)

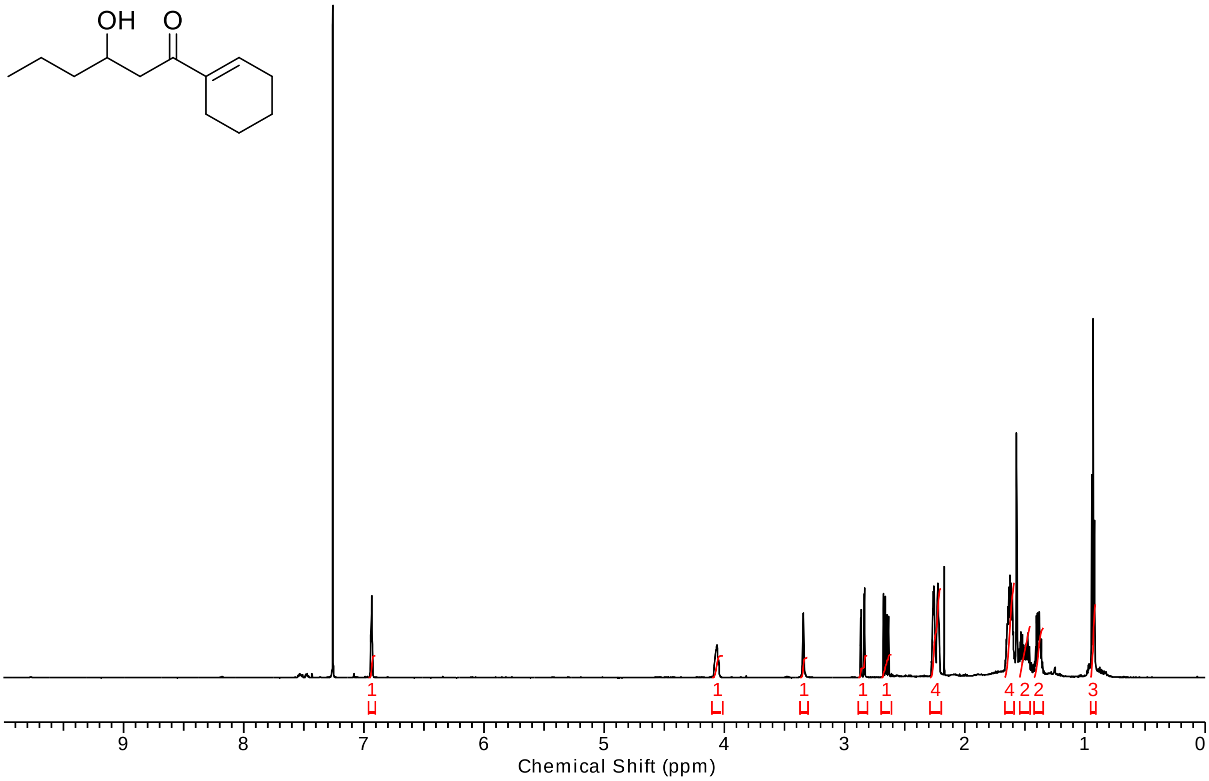

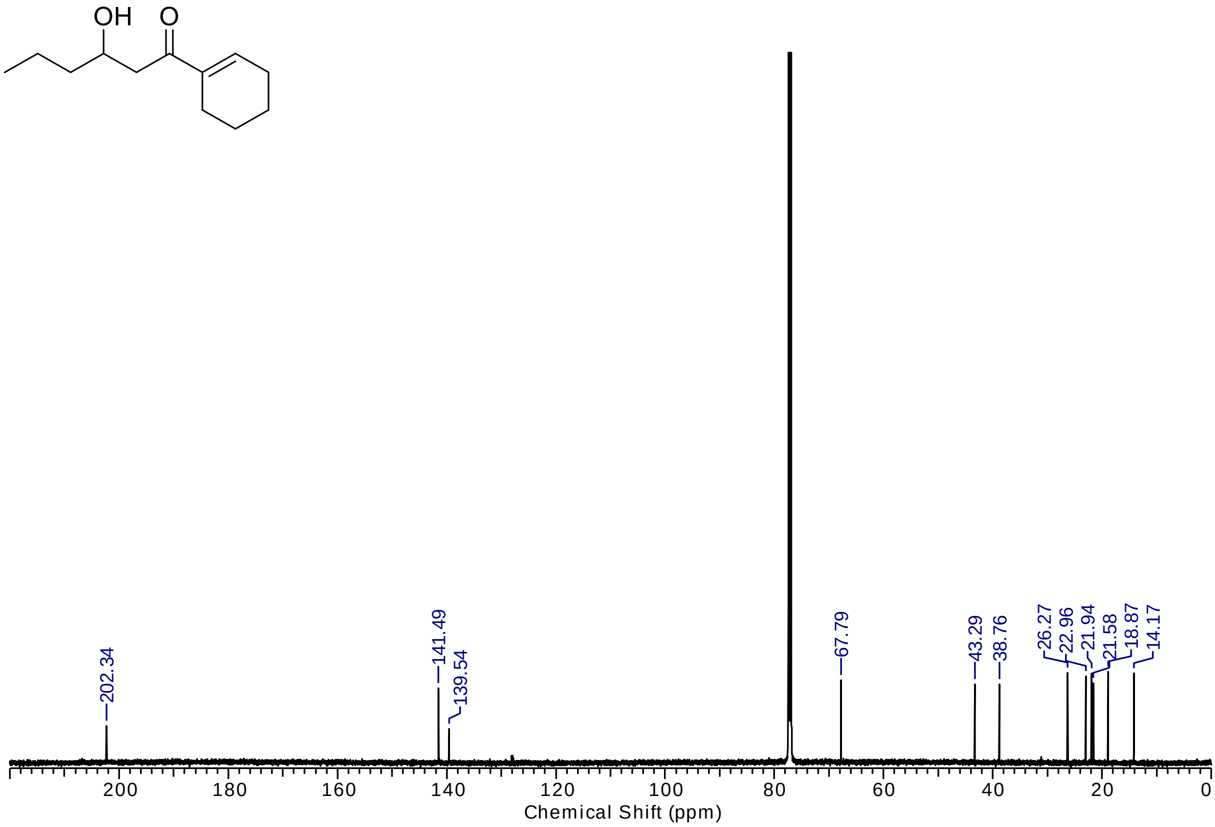

3-Hydroxy-1-(4-methoxyphenyl)-5-phenylpentan-1-one (7g)

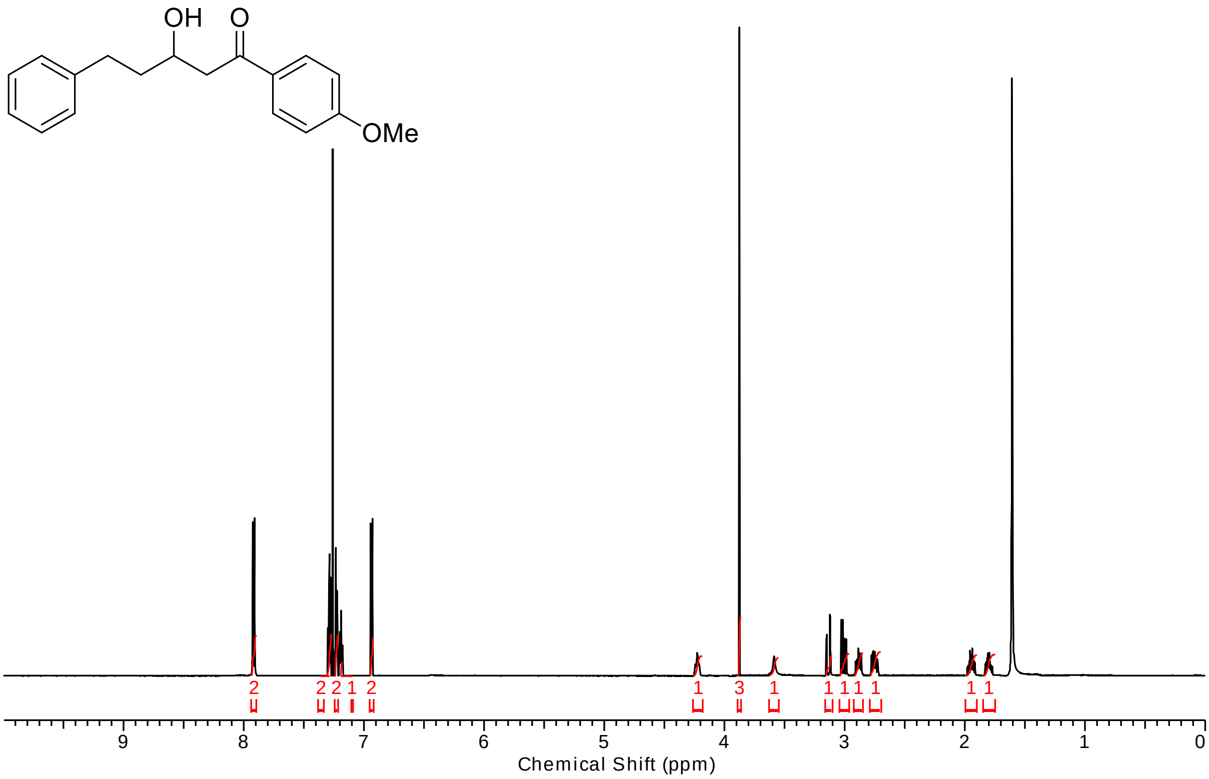

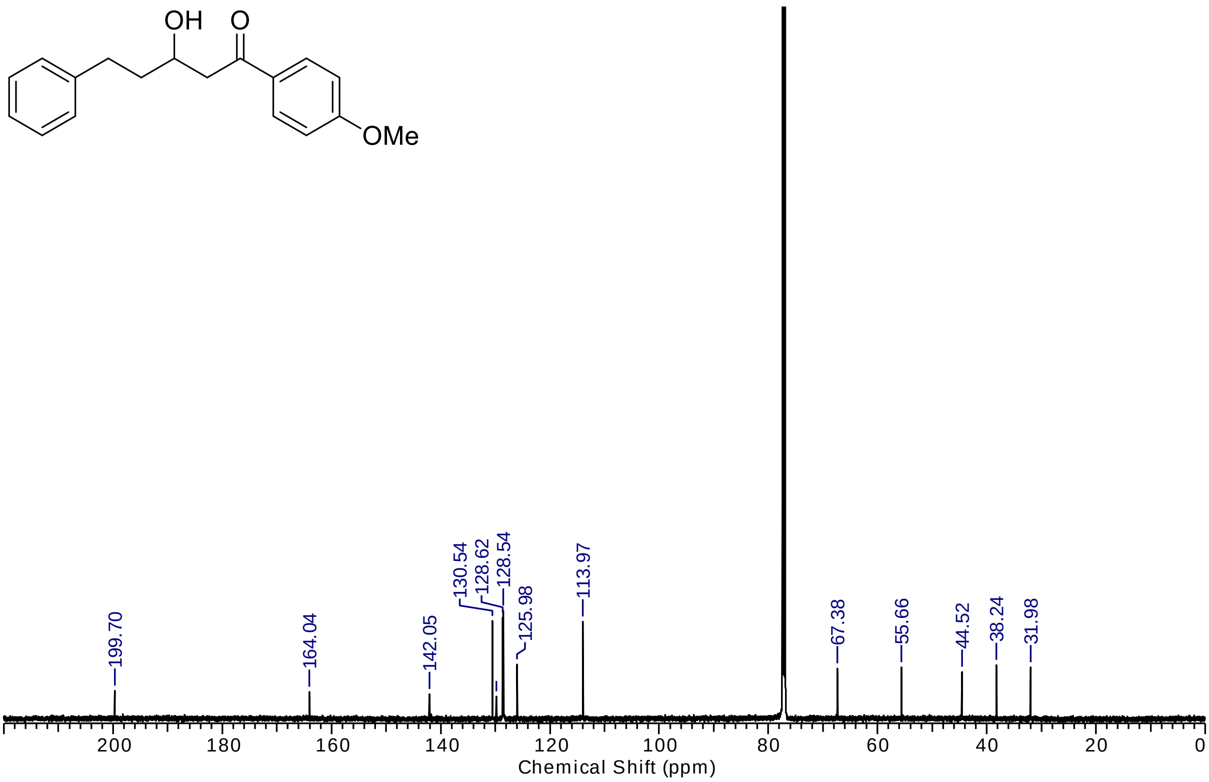

1-Cyclopentyl-3-hydroxy-5-methylhexan-1-one (7h)

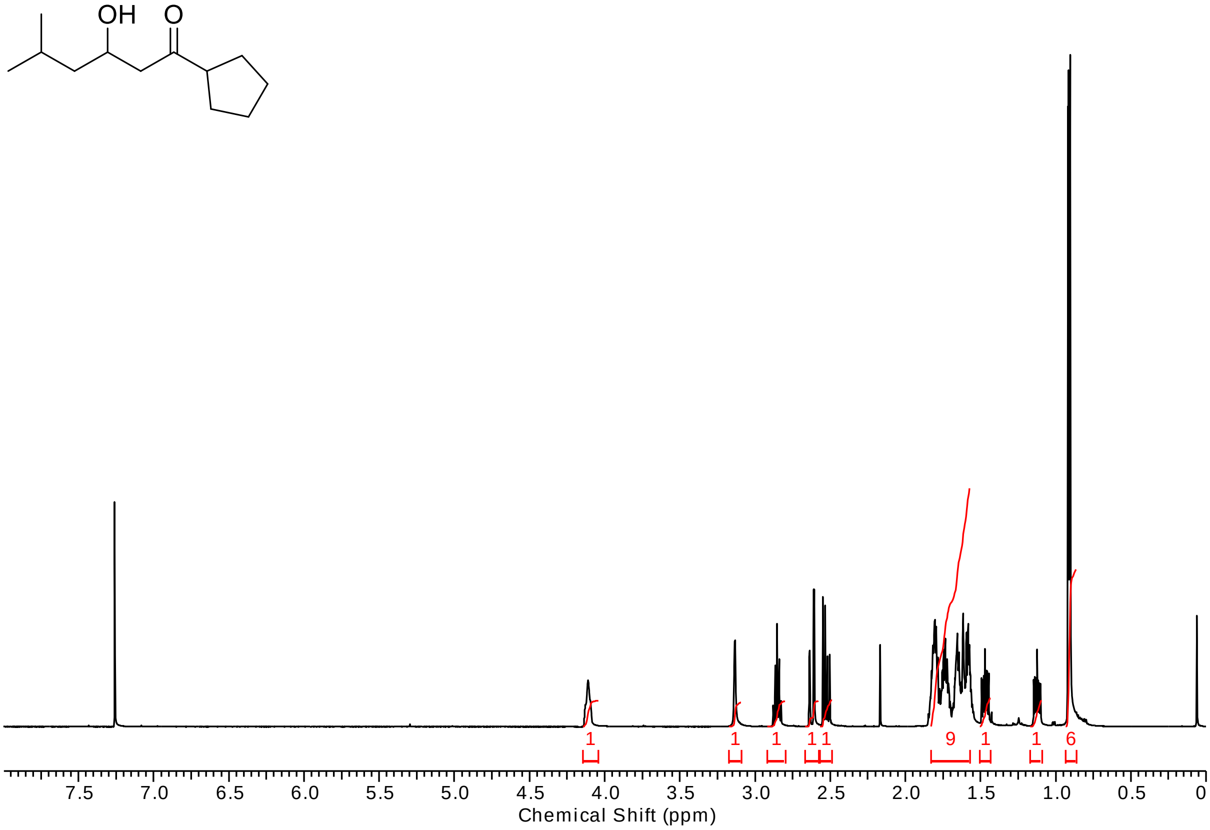

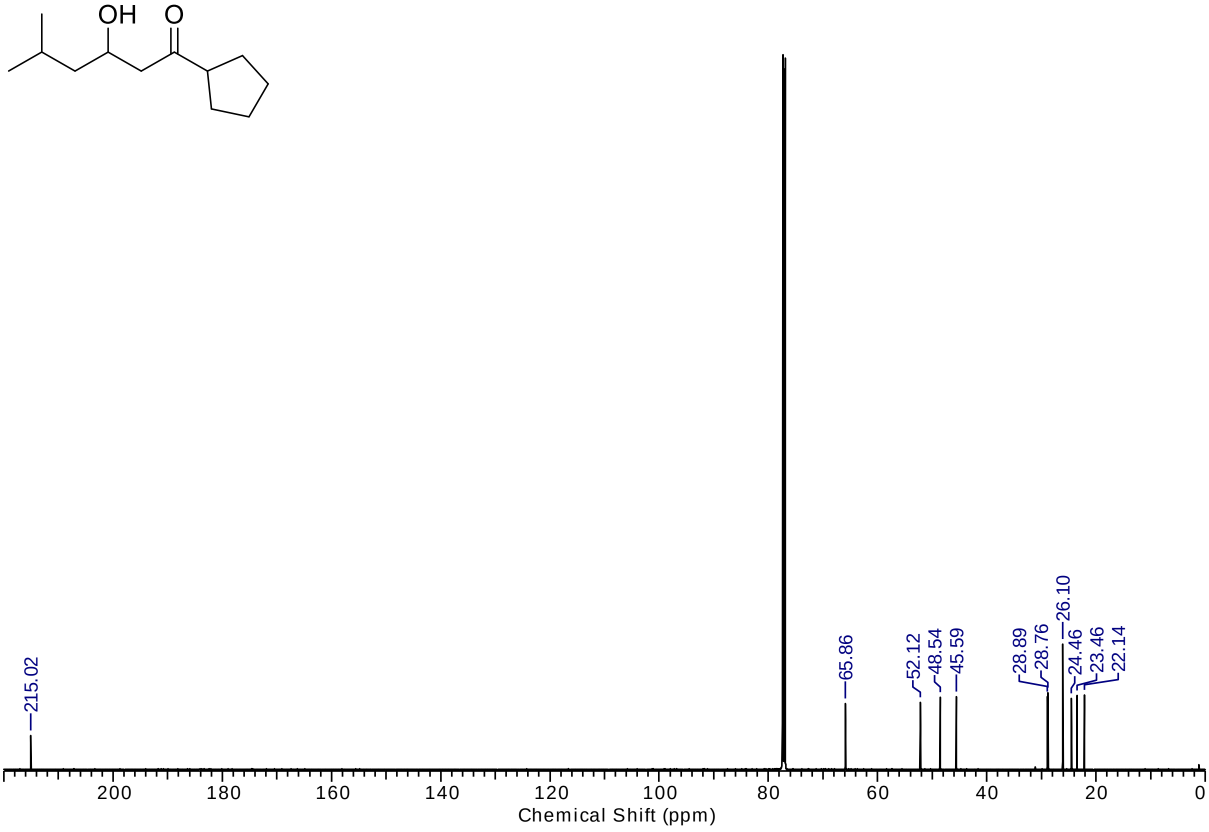

1,1,1-Trifluoro-2-hydroxy-2-phenyl-octan-4-one (7i)

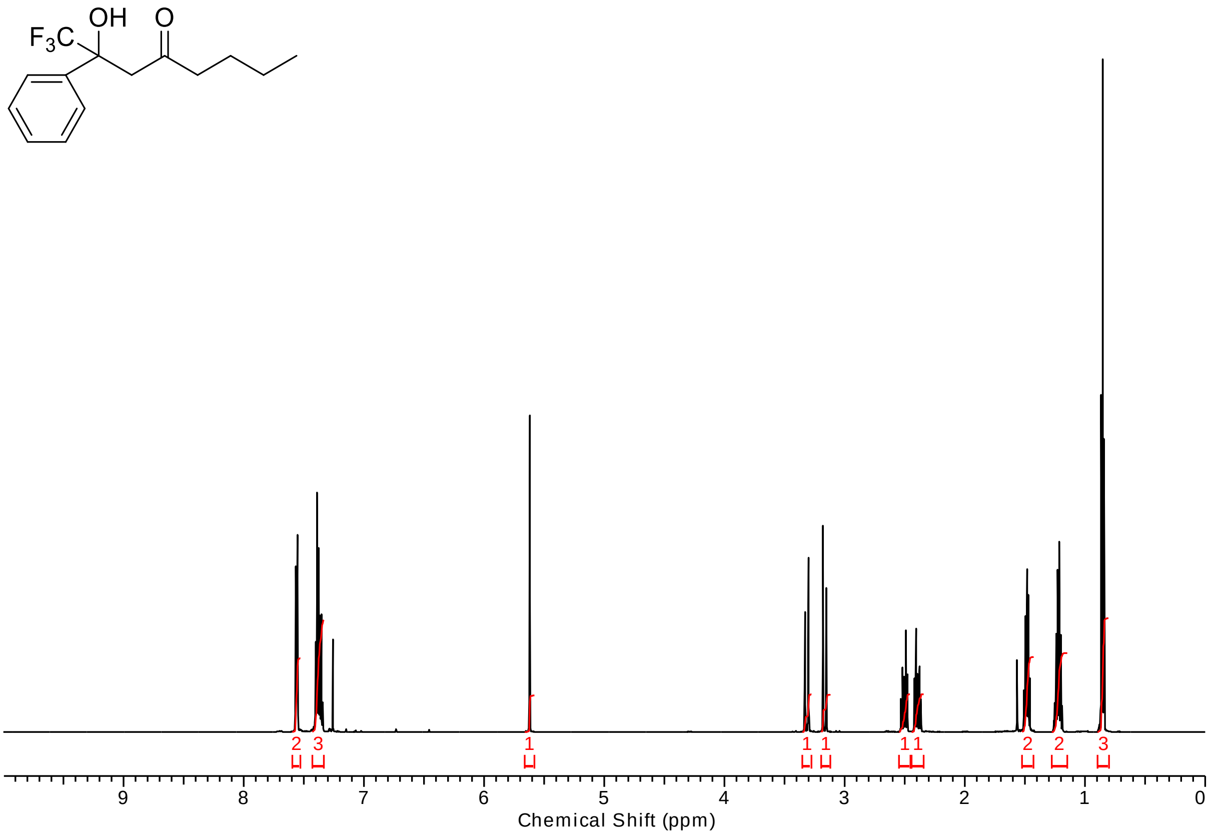

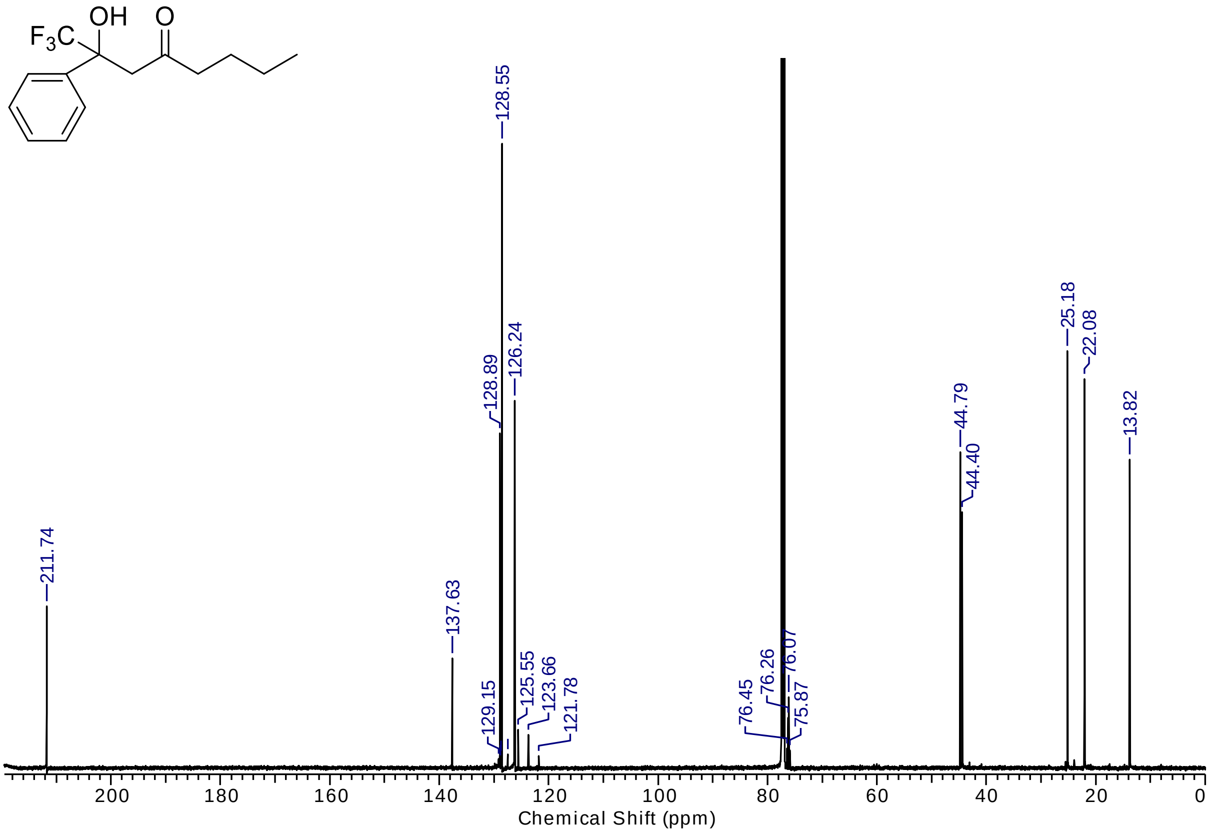

1,1,1-Trifluoro-2-hydroxy-2-methyl-octan-4-one (7j)

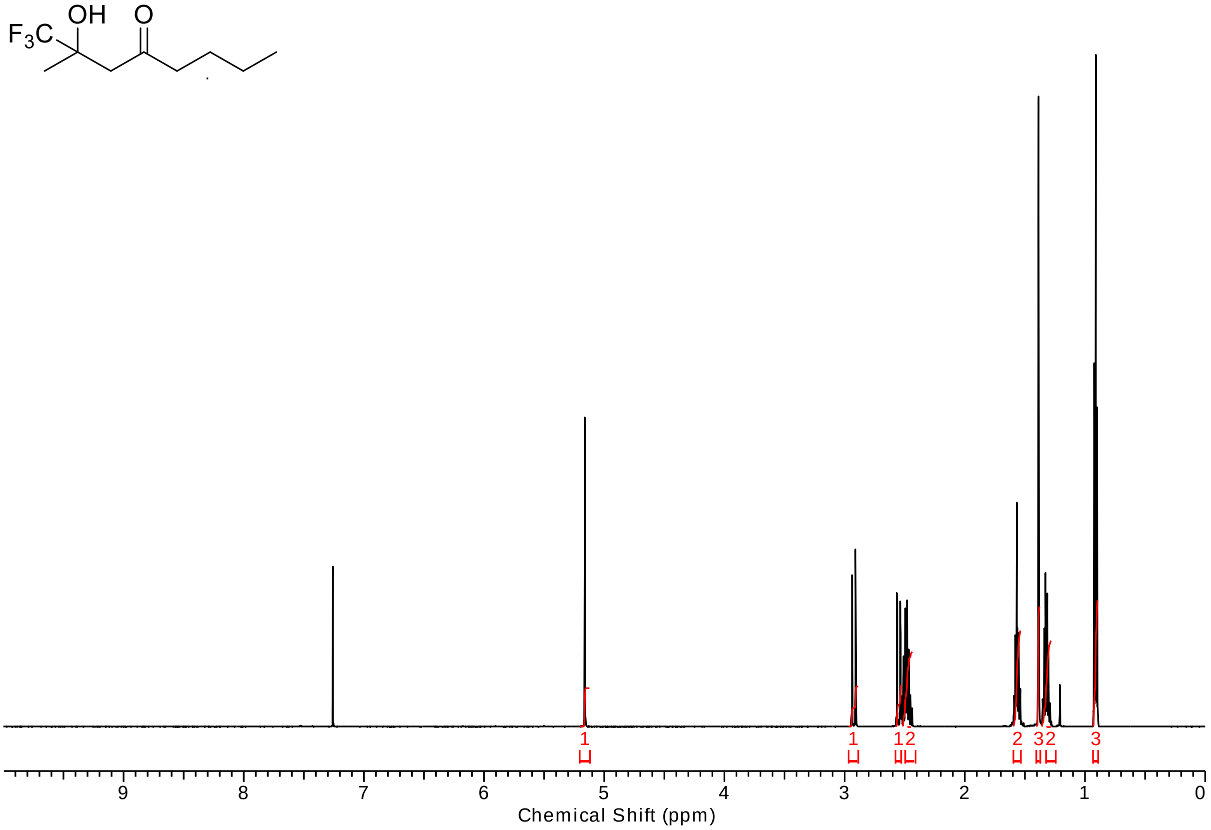

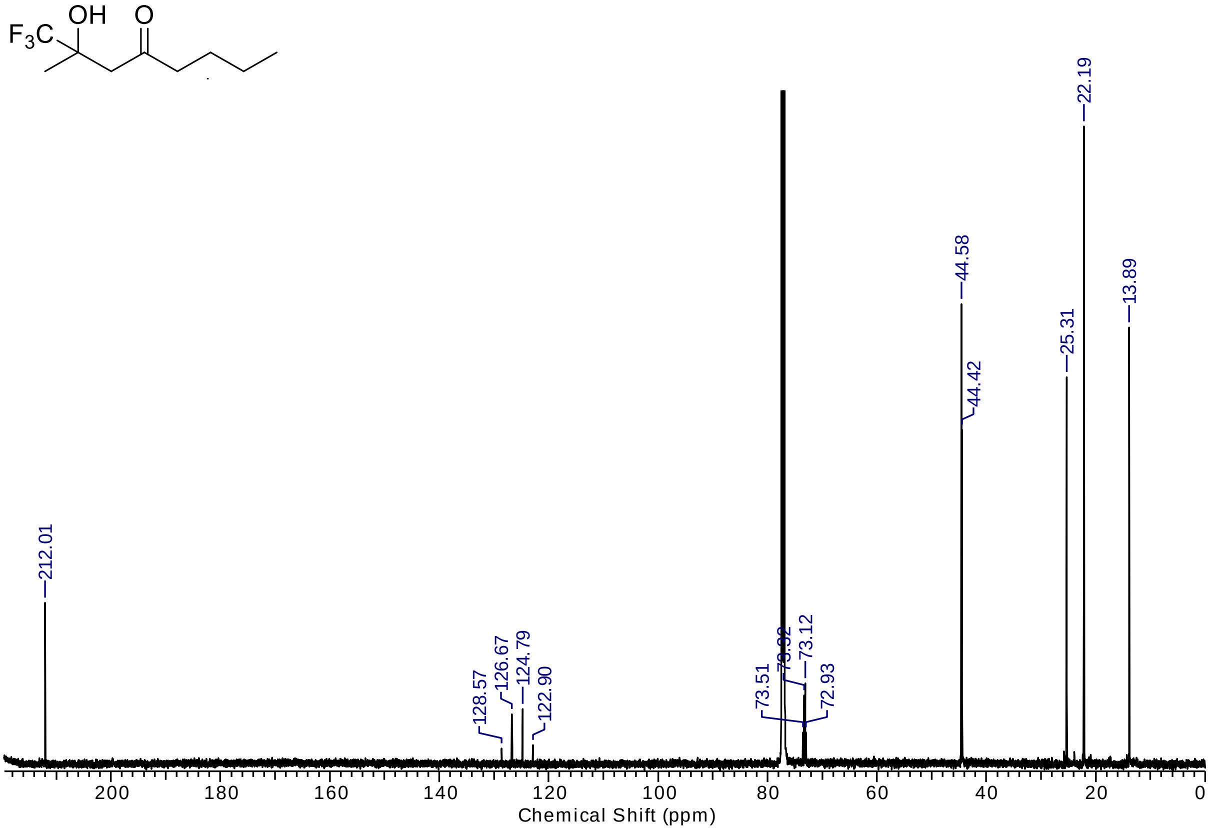

3-Hydroxy-4-methyl-1-phenylpentan-1-one (*S*)-7o

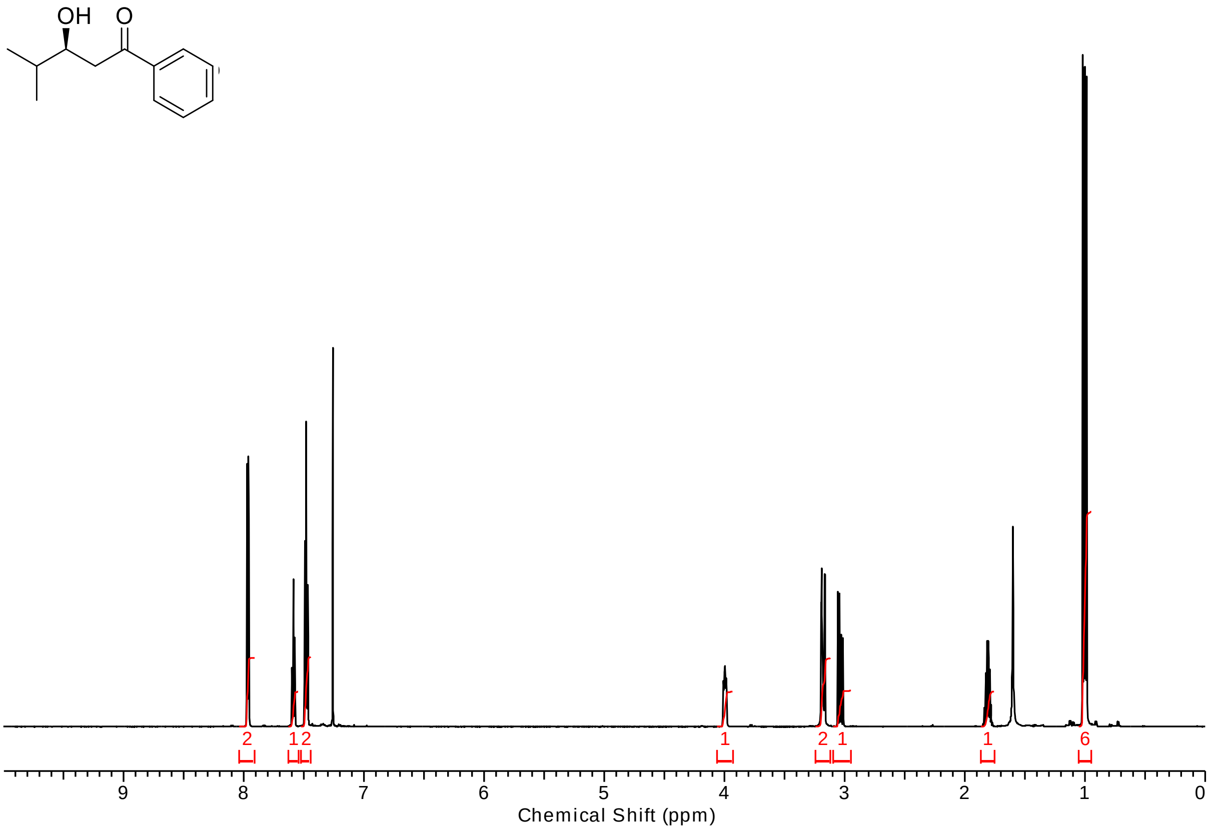

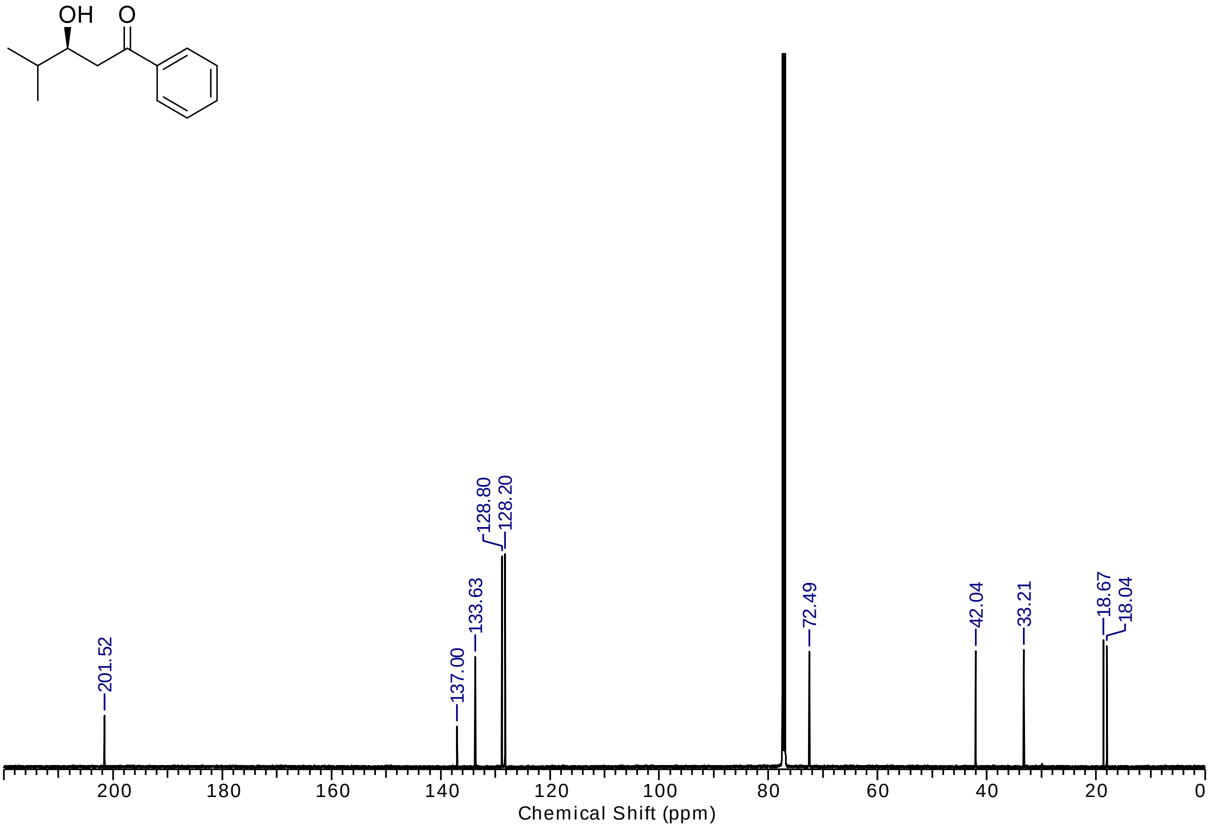

(±)-7o

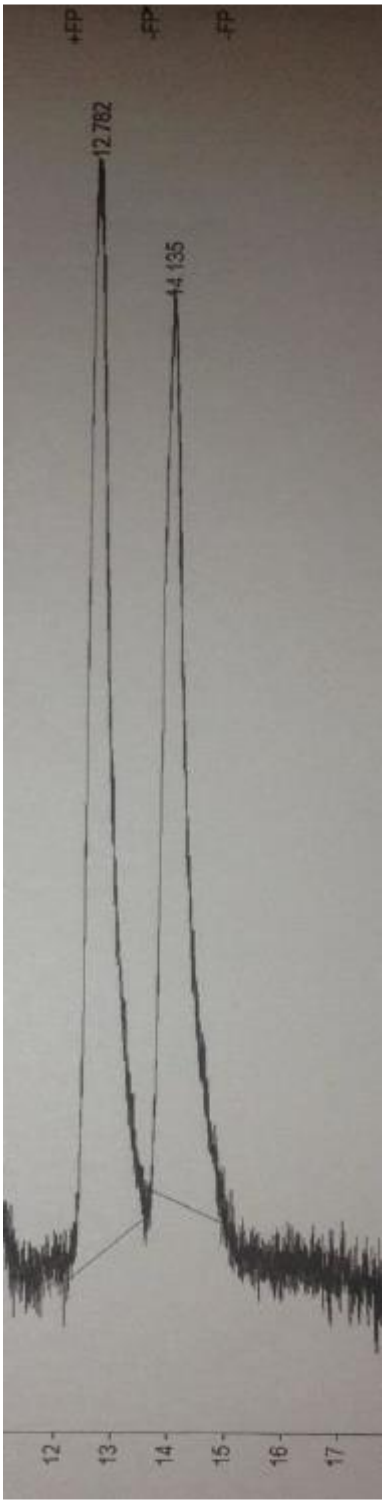

(*S*)-7o

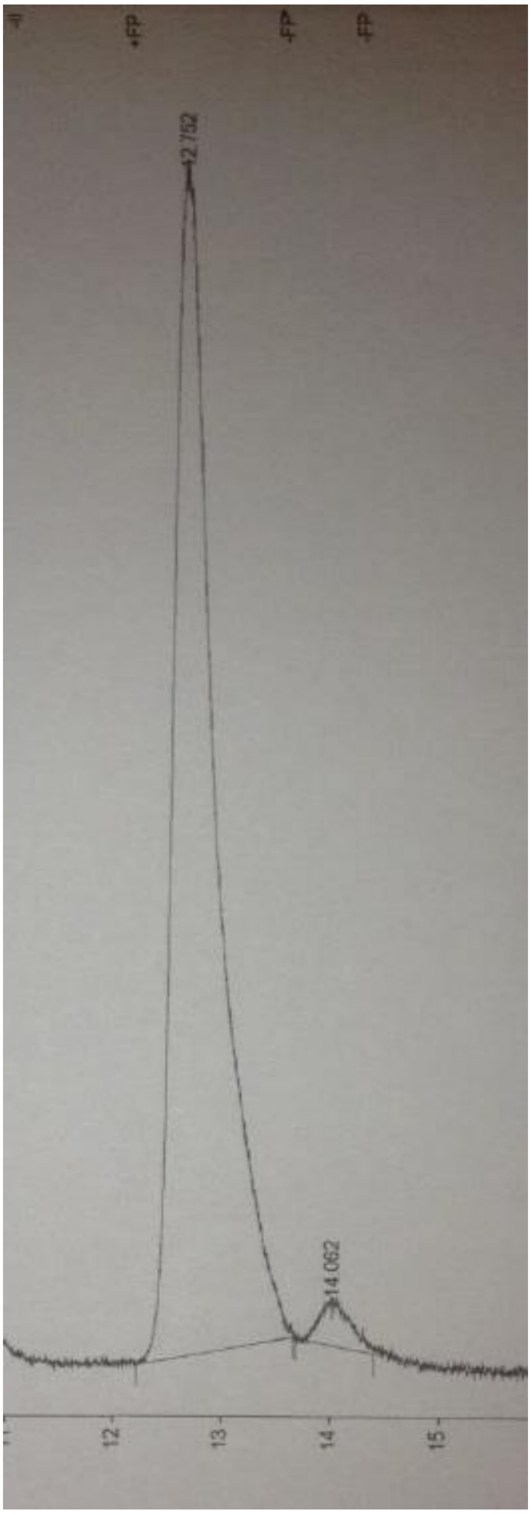

Boron Enolate 5p

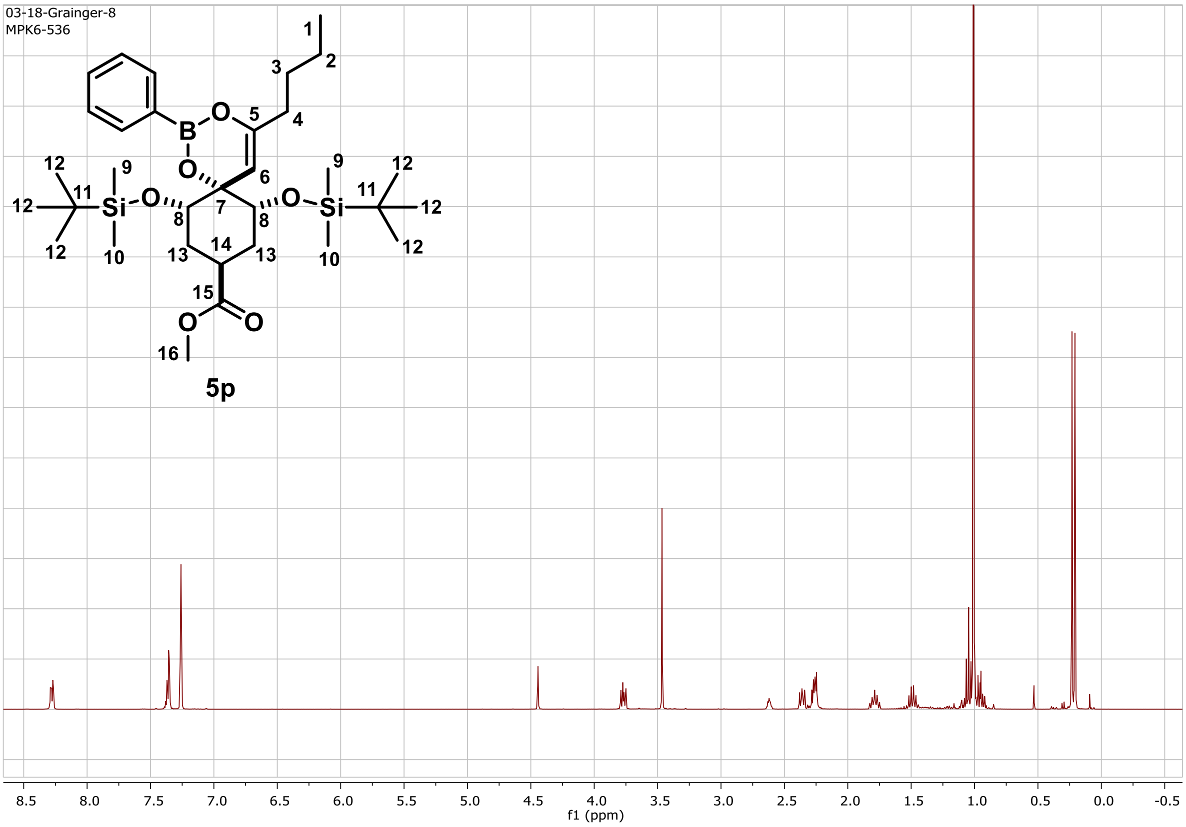

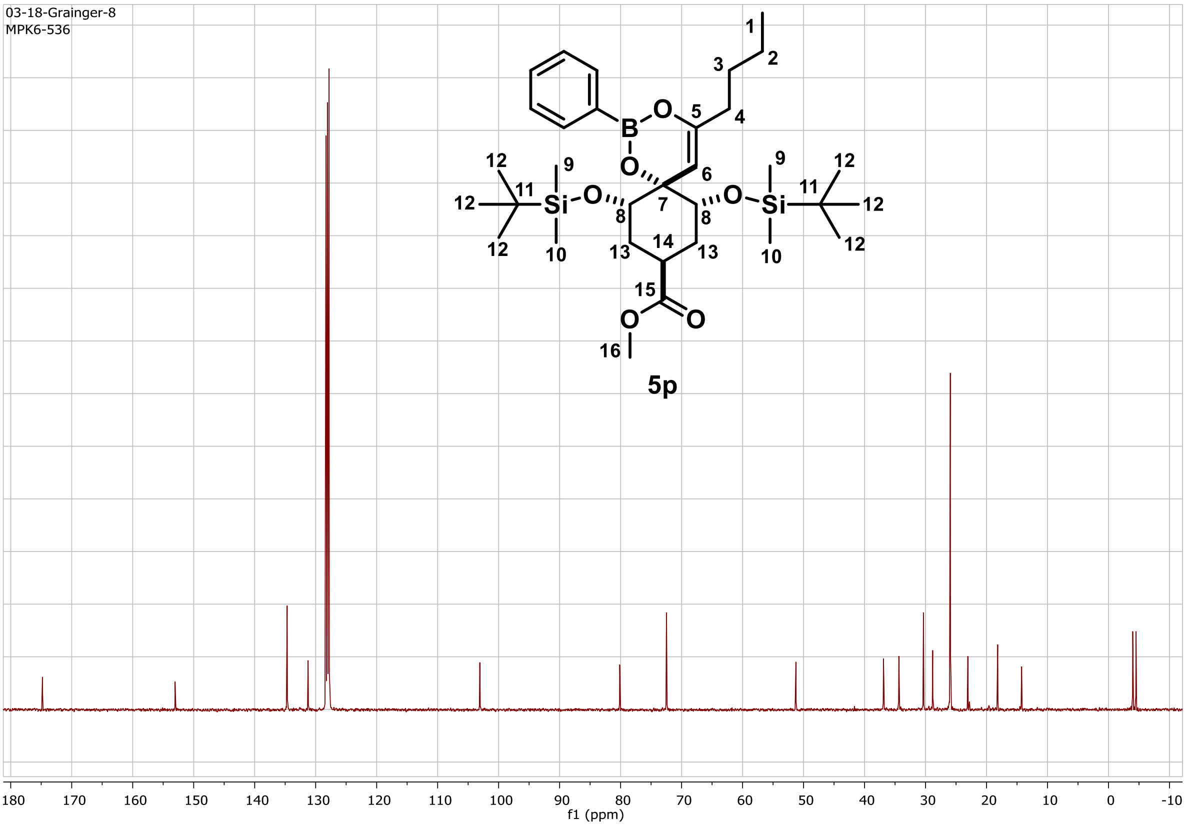

Hydroxyketone 7p

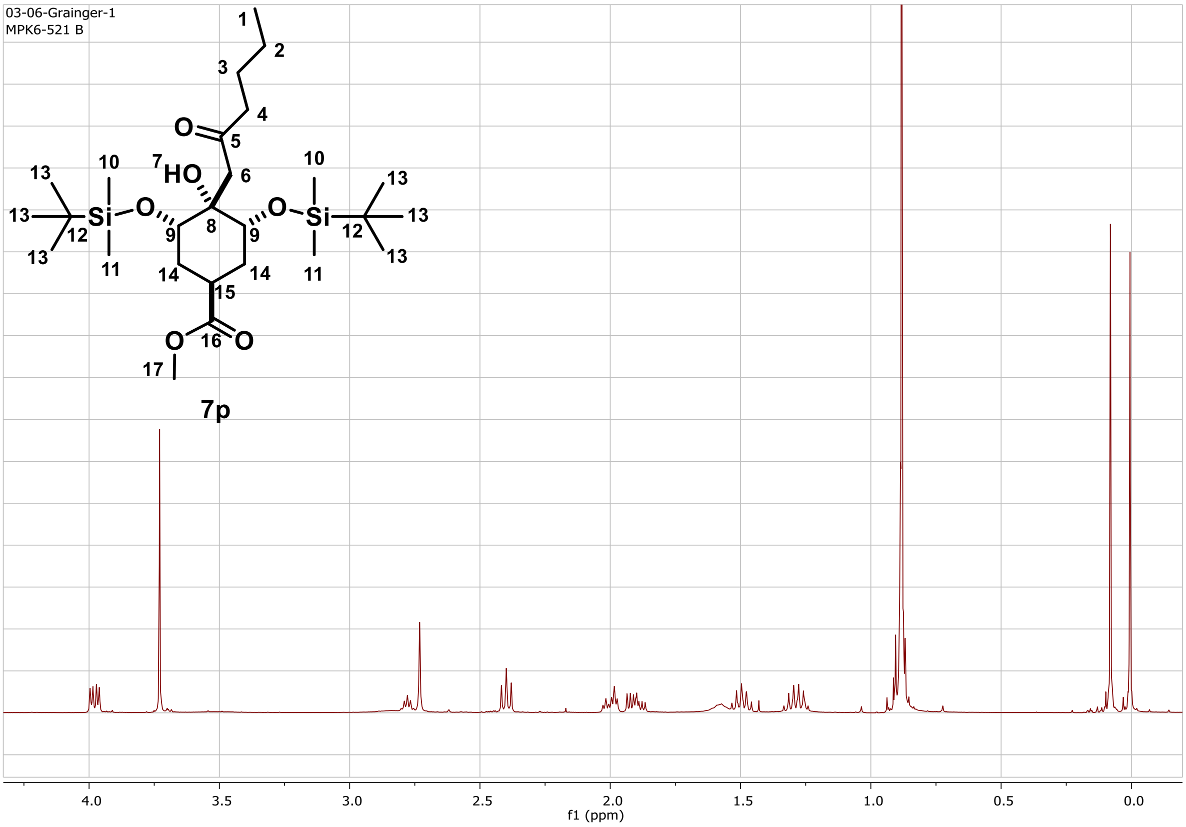

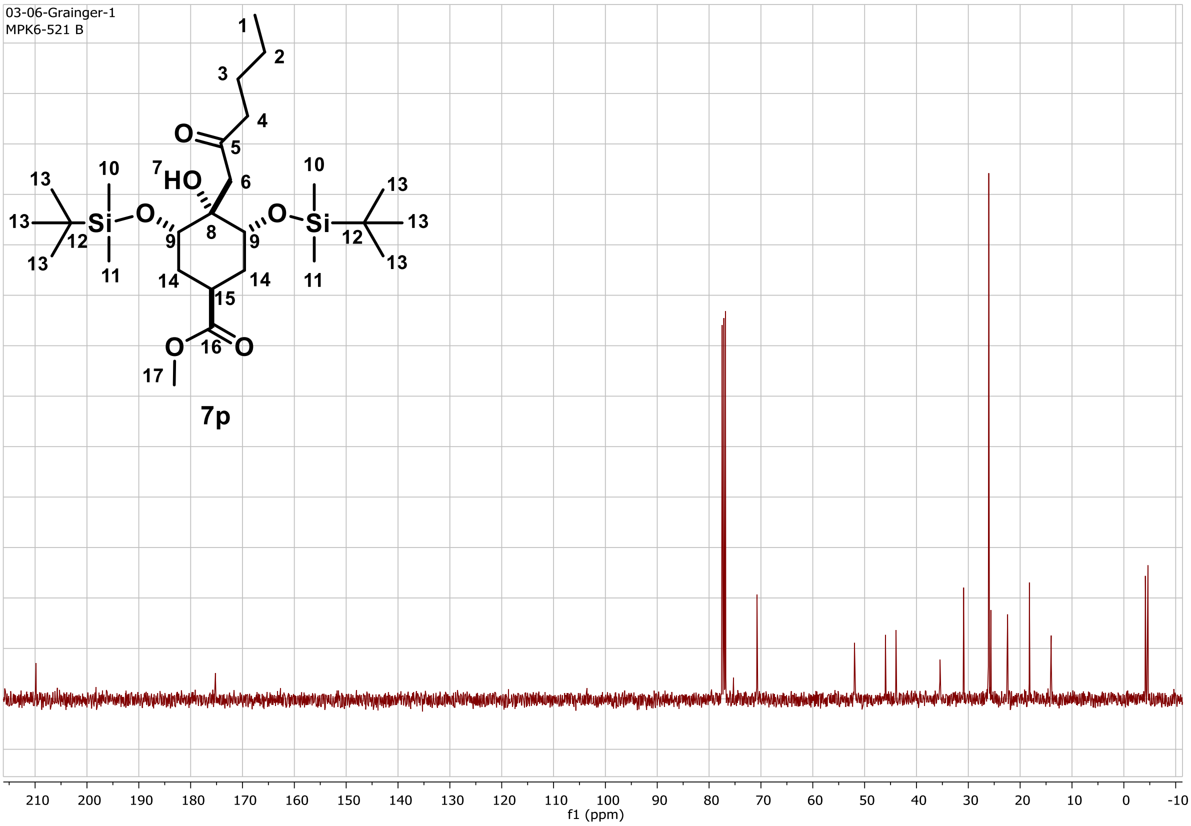

5. X-ray Crystallography data for Boron Enolate 5p

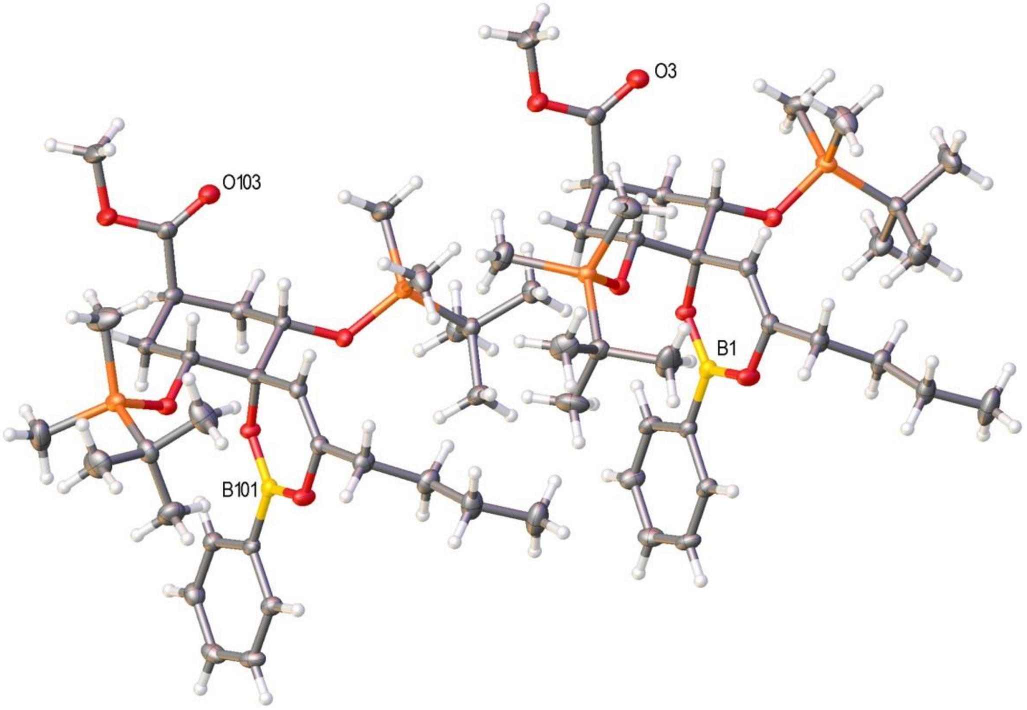

Figure S1: Crystal structure of **5p** with ellipsoids drawn at the 50 % probability level. The structure contains two crystallographically-independent molecules.

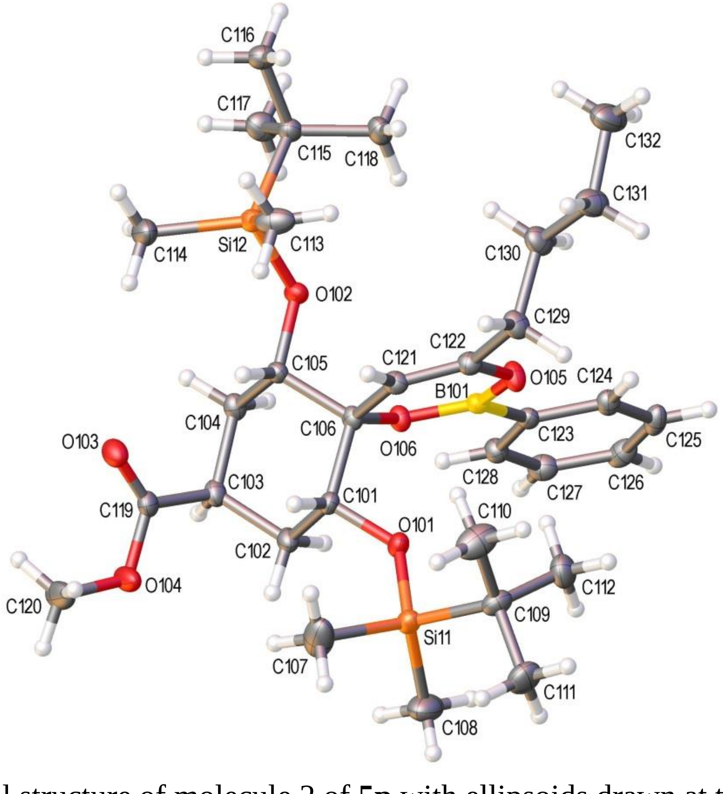

Figure S2: Crystal structure of molecule 2 of **5p** with ellipsoids drawn at the 50 % probability level. The structure contains two crystallographically-independent molecules of which only one is shown.

Crystal Data for 5p $C_{32}H_{55}BO_6Si_2$ (M =602.75 g/mol): triclinic, space group P-1 (no. 2), a = 11.2926(3) Å, b = 17.6773(4) Å, c = 19.8735(5) Å, α = 101.118(2)°, β = 98.960(2)°, γ = 107.432(2)°, V = 3616.08(17) Å^3, Z = 4, T = 99.97(17) K, μ(CuKα) = 1.186 mm^{-1}, $Dcalc$ = 1.107 g/cm^3, 49473 reflections measured (5.418° ≤ 2Θ ≤ 140.126°), 13726 unique (R_{int} = 0.0328, R_{sigma} = 0.0322) which were used in all calculations. The final R_1 was 0.0405 (I > 2σ(I)) and wR_2 was 0.1045 (all data).

The dataset was measured on an Agilent SuperNova diffractometer using an Atlas detector. The data collection was driven and processed and an absorption correction was applied using CrysAlisPro.[7] The structure was solved using ShelXS [8] and refined by a full-matrix least-squares procedure on F^2 in ShelXL.[9] All non-hydrogen atoms were refined with anisotropic displacement parameters. All hydrogen atoms were added at calculated positions and refined by use of a riding model with isotropic displacement parameters based on the equivalent isotropic displacement parameter (U_{eq}) of the parent atom. Figures and reports were produced using OLEX2.[S3] The CIF for the crystal structure of **5p** has been deposited with the CCDC and have been given the deposition number CCDC 1448535.

.

6. References

[1] M. N. Pennell, P. G. Turner, T. D. Sheppard, *Chem. Eur. J.* **2012**, *18*, 4748; M. N. Pennell, M. G. Unthank, P. Turner, *J. Org. Chem.* **2011**, *76*, 1479.

[2] C. A. Correia, D. T. McQuade, P. H. Seeberger, *Adv. Synth. Catal.* **2013**, *355*, 3517.

[3] D. E. Frantz, R. Fässler, E. M. Carreira, *J. Am. Chem. Soc.* **2000**, *122*, 1806-1807; D. Boyall, D. E. Frantz, E. M. Carreira, *Org. Lett.* **2002**, *4*, 2605.

[4] C. Roche, O. Labeeuw, M. Haddad, T. Ayad, J. -P. Genet, V. Ratovelomanana-Vidal, P. Phansavath, *Eur. J. Org. Chem.* **2009**, 3977.

[5] S. Kogiso, S. Hosozawa, K. Wada, K. Munakata, *Phytochemistry* **1974**, *13*, 2332.

[6] S. Kobayashi, P. Xu, T. Endo, M. Ueno, T. Kitanoso, *Angew. Chem. Int. Ed.* **2012**, *51*, 1276.

[7] CrysAlisPro, Agilent Technologies, Version 1.171.36.28, **2013**.

[8] G. M. Sheldrick, *Acta Cryst.* **2008**, *A64*, 112-122.

[9] O. V. Dolomanov, L. J. Bourhis, R. J. Gildea, J. A. K. Howard, H. Puschmann, *J. Appl. Crystallogr.* **2009**, *42*, 339-341.
